# Discovery of novel mRNA demethylase FTO inhibitors against esophageal cancer

**DOI:** 10.1080/14756366.2022.2098954

**Published:** 2022-07-14

**Authors:** Bo Qin, Qian Bai, Dan Yan, Fanxiang Yin, Zhu Zhu, Chaoyuan Xia, Yang Yang, Yi Zhao

**Affiliations:** a Translational Medical Center, The First Affiliated Hospital of Zhengzhou University, Zhengzhou, PR China; b Department of Anesthesiology, The Second Affiliated Hospital of Zhengzhou University, Zhengzhou, PR China

**Keywords:** FTO, 1,2,3-triazole, esophageal cancer, cell cycle, molecular docking

## Abstract

A series of 1,2,3-triazole analogues as novel fat mass and obesity-associated protein (FTO) inhibitors were synthesised in this study. Among all 1,2,3-triazoles, compound **C6** exhibited the most robust inhibition of FTO with an IC_50_ value of 780 nM. It displayed the potent antiproliferative activity against KYSE-150, KYSE-270, TE-1, KYSE-510, and EC109 cell lines with IC_50_ value of 2.17, 1.35, 0.95, 4.15, and 0.83 μM, respectively. In addition, **C6** arrested the cell cycle at G2 phase against TE-1 and EC109 cells in a concentration-dependent manner. Analysis of cellular mechanisms demonstrated that **C6** concentration-dependently regulated epithelial mesenchymal transition (EMT) pathway and PI3K/AKT pathway against TE-1 and EC109 cells. Molecular docking studies that **C6** formed important hydrogen-bond interaction with Lys107, Asn110, Tyr108, and Leu109 of FTO. These findings suggested that **C6** as a novel FTO inhibitor and orally antitumor agent deserves further investigation to treat esophageal cancer.

## GRAPHICAL ABSTRACT

**Figure:**
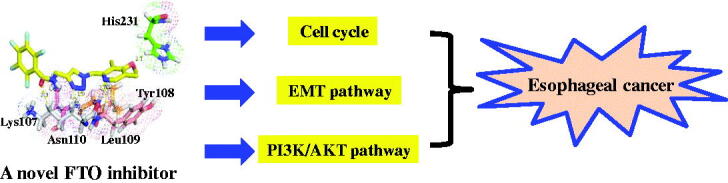

## 1. Introduction

Esophageal cancer as the eighth most common cancer in the world possesses the poor prognosis and poor survival^1^. It is necessary to develop effective and novel drugs to treat esophageal cancer^2^. Fat mass and obesity-associated protein (FTO), a demethylase for *N*^6^-methyladenosine modification, has been implicated in esophageal cancer^3^. Recent report demonstrated that esophageal cancer tissues had the increased FTO expression which correlated with clinical esophageal cancer prognosis^4^. In addition, FTO could play oncogenic roles and promote cell proliferation and migration in esophageal cancer^5^. Therefore, FTO might be a potential target and FTO inhibitors might be effective and novel anticancer agents for the treatment of esophageal cancer.

**Figure 1.**
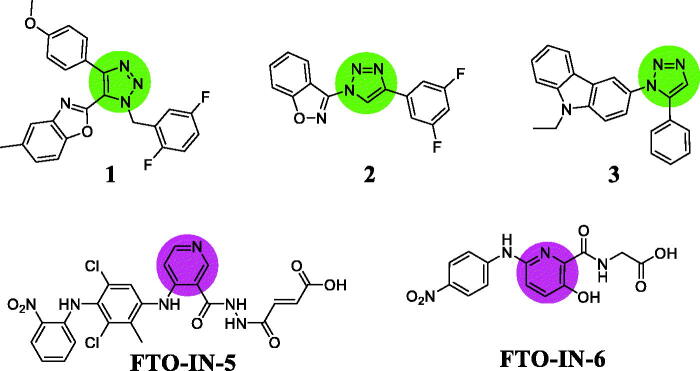
1,2,3-Triazole hybrids and pyridine-based FTO inhibitors.

1,2,3-Triazole as one of the most important classes of nitrogen-containing heterocycle exhibits potent anticancer activity^6^. 1,2,3-triazole-benzoxazole hybrid **1** (Figure 1) displayed antiproliferative activity against SKBr3, HepG2, and HeLa cells with IC_50_ values of 7.1, 11.2, and 6.8 μg/mL^7^. 1,2,3-Triazole-benzisoxazole hybrid **2** showed antiproliferative activity against MOLM13, MOLM14, and MV4-11 cell lines^8^. Hybrid **3** inhibited migration and mammosphere formation and induced cell cycle arrest at G2-M phase against breast cancer cells^9^. On the other hand, pyridine derivatives also have a wide-range of therapeutic applications in the area of drug discovery^10^. Pyridine analogue FTO-IN-5 (Figure 1) as a selective FTO inhibitor could decrease the viability of acute monocytic leukaemia cells and increase the level of *N*^6^-methyladenosine in mRNA^11^. Pyridine analogue FTO-IN-6 selectively inhibited FTO and formed hydrogen bonds with residues Ser318 and Tyr295^12^.

Molecular hybridisation, involving a combination of two or more bioactive scaffolds to generate a single molecular architecture, has been a promising strategy in the drug discovery research^13^. Furthermore, fluorine as the most electronegative element plays a key role to design anticancer agents^14^. Therefore, a class of 1,2,3-triazole-pyridine hybrids containing a pentafluorobenzoyl moiety as potential FTO inhibitors was designed by the molecular hybridisation. In addition, these compounds were evaluated for their anticancer activity *in vitro* and *in vivo* against esophageal cancer cell lines. To the best of our knowledge, it is the first time to discover that 1,2,3-triazole-pyridine hybrids could be potential anticancer agents by targeting FTO for the treatment of esophageal cancer.

## 2. Materials and methods

### 2.1. Chemistry experimental procedures

Chemical reagents and organic solvents were purchased from commercial sources (Innochem, Beijing, China). Melting points of all compounds were measured using a melting point detector (Tianjin Jingtuo Instrument Technology Co., LTD, Tianjin, China). ^1^HNMR and ^13^CNMR spectrum of all new compounds were recorded on a Bruker spectrometer (Bruker, Karlsruhe, Germany). High-resolution mass spectra were recorded on a Waters Micromass spectrometer (Waters, Shanghai, China).

### 2.2. Synthesis of compound B

A mixture of 2,3,4,5,6-pentafluorobenzoyl chloride **A** (5 mmol), triethylamine (6 mmol), and prop-2-yn-1-amine (6 mmol) was refluxed in dichloromethane (25 ml) for 6 h. Upon completion, the reaction system was washed with dichloromethane to afford the crude product. The crude product was purified by silica gel chromatography (PE:EA = 7:1) to yield compound **B**.

### 2.3. Synthesis of compound C1∼C8

The mixture of compound **B** (1 mmol), azide derivatives (1 mmol), copper sulphate pentahydrate (0.2 mmol), and sodium ascorbate (0.2 mmol) in tetrahydrofuran (10 ml) and water (10 ml) was stirred at 25 °C for 12 h. After the filtration and vacuum concentration, residues were purified by silica gel chromatography (PE:EA = 9:1) to generate compound **C1∼C8**.

#### 2.3.1. 2,3,4,5,6-Pentafluoro-N-(prop-2-yn-1-yl)benzamide (B)

White solid, yield: 79%. Mp: 83–85 °C. ^1^H NMR (400 MHz, DMSO-d_6_) δ 9.40 (s, 1H), 4.10 (dd, *J* = 5.5, 2.5 Hz, 2H), 3.21 (s, 1H). ^13 ^C NMR (100 MHz, DMSO-d_6_) δ 156.41, 144.34, 141.87, 139.98, 138.16, 135.66, 111.95, 79.78, 73.67, 28.64. HR-MS (ESI): calcd for C_10_H_5_F_5_NO, [M + H]^+^ 250.0291; found: 250.0297. Purity: 96.82%.

#### 2.3.2. N-((1-((2-chloropyridin-3-yl)methyl)-1H-1,2,3-triazol-4-yl)methyl)-2,3,4,5,6-pentafluorobenzamide (C1)

White solid, yield: 83%. Mp: 152–154 °C. ^1^H NMR (400 MHz, DMSO-d_6_) δ 9.44 (t, *J* = 5.4 Hz, 1H), 8.45 (d, *J* = 2.3 Hz, 1H), 8.13 (s, 1H), 7.81 (dd, *J* = 8.3, 2.5 Hz, 1H), 7.55 (d, *J* = 8.2 Hz, 1H), 5.69 (s, 2H), 4.53 (d, *J* = 5.6 Hz, 2H). ^13 ^C NMR (100 MHz, DMSO-d_6_) δ 156.55, 150.08, 149.48, 144.01, 139.57, 131.36, 124.41, 123.34, 49.37, 34.87. HR-MS (ESI): calcd for C_16_H_10_ClF_5_N_5_O, [M + H]^+^ 418.0494; found: 418.0498. Purity: 97.43%.

#### 2.3.3. N-((1-((6-chloropyridin-3-yl)methyl)-1H-1,2,3-triazol-4-yl)methyl)-2,3,4,5,6-pentafluorobenzamide (C2)

White solid, yield: 80%. Mp: 148–150 °C. ^1^H NMR (400 MHz, DMSO-d_6_) δ 9.44 (t, *J* = 5.4 Hz, 1H), 8.45 (d, *J* = 2.3 Hz, 1H), 8.14 (s, 1H), 7.81 (dd, *J* = 8.3, 2.5 Hz, 1H), 7.55 (d, *J* = 8.2 Hz, 1H), 5.69 (s, 2H), 4.53 (d, *J* = 5.6 Hz, 2H). ^13 ^C NMR (100 MHz, DMSO-d_6_) δ 156.55, 150.08, 149.48, 144.01, 139.57, 131.35, 124.40, 123.33, 49.37, 34.87. HR-MS (ESI): calcd for C_16_H_10_ClF_5_N_5_O, [M + H]^+^ 418.0494; found: 418.0498. Purity: 97.57%.

#### 2.3.4. 2,3,4,5,6-Pentafluoro-N-((1-(pyridin-4-ylmethyl)-1H-1,2,3-triazol-4-yl)methyl)benzamide (C3)

White solid, yield: 72%. Mp: 108–110 °C. ^1^H NMR (400 MHz, DMSO-d_6_) δ 9.49 (t, *J* = 5.4 Hz, 1H), 8.57 (d, *J* = 3.8 Hz, 2H), 8.16 (s, 1H), 7.21 (d, *J* = 5.6 Hz, 2H), 5.72 (s, 2H), 4.58 (d, *J* = 5.6 Hz, 2H). ^13 ^C NMR (100 MHz, DMSO-d_6_) δ 156.58, 149.96, 144.90, 144.02, 123.76, 122.24, 51.44, 34.89. HR-MS (ESI): calcd for C_16_H_11_F_5_N_5_O, [M + H]^+^ 384.0884; found: 384.0889. Purity: 98.06%.

#### 2.3.5. 2,3,4,5,6-Pentafluoro-N-((1-(pyridin-2-ylmethyl)-1H-1,2,3-triazol-4-yl)methyl)benzamide (C4)

White solid, yield: 88%. Mp: 149–151 °C. ^1^H NMR (400 MHz, DMSO-d_6_) δ 9.47 (t, *J* = 5.3 Hz, 1H), 8.55 (d, *J* = 4.2 Hz, 1H), 8.08 (s, 1H), 7.83 (td, *J* = 7.7, 1.7 Hz, 1H), 7.60 − 7.09 (m, 2H), 5.73 (s, 2H), 4.56 (d, *J* = 5.6 Hz, 2H). ^13 ^C NMR (100 MHz, DMSO-d_6_) δ 156.55, 155.02, 149.40, 143.68, 137.27, 123.78, 123.20, 122.14, 54.34, 34.90. HR-MS (ESI): calcd for C_16_H_11_F_5_N_5_O, [M + H]^+^ 384.0884; found: 384.0887. Purity: 96.99%.

#### 2.3.6. 2,3,4,5,6-Pentafluoro-N-((1-((3-methyl-4–(2,2,2-trifluoroethoxy)pyridin-2-yl)methyl)-1H-1,2,3-triazol-4-yl)methyl)benzamide (C5)

White solid, yield: 69%. Mp: 156–158 °C. ^1^H NMR (400 MHz, DMSO-d_6_) δ 9.44 (t, *J* = 5.5 Hz, 1H), 8.30 (d, *J* = 5.6 Hz, 1H), 7.92 (s, 1H), 7.13 (d, *J* = 5.7 Hz, 1H), 5.74 (s, 2H), 4.92 (q, *J* = 8.7 Hz, 2H), 4.54 (d, *J* = 5.6 Hz, 2H), 2.22 (s, 3H). ^13 ^C NMR (100 MHz, DMSO-d_6_) δ 161.30, 156.55, 153.95, 148.03, 143.45, 123.62, 120.06, 107.33, 64.82, 64.48, 52.43, 34.90, 9.70. HR-MS (ESI): calcd for C_19_H_14_F_8_N_5_O_2_, [M + H]^+^ 496.1020; found: 496.1025. Purity: 97.43%.

#### 2.3.7. 2,3,4,5,6-Pentafluoro-N-((1-((4-methoxy-3,5-dimethylpyridin-2-yl)methyl)-1H-1,2,3-triazol-4-yl)methyl)benzamide (C6)

White solid, yield: 62%. Mp: 135–137 °C. ^1^H NMR (400 MHz, DMSO-d_6_) δ 9.44 (t, *J* = 5.3 Hz, 1H), 8.18 (s, 1H), 7.90 (s, 1H), 5.69 (s, 2H), 4.53 (d, *J* = 5.6 Hz, 2H), 3.73 (s, 3H), 2.23 (d, *J* = 19.3 Hz, 6H). ^13 ^C NMR (100 MHz, DMSO-d_6_) δ 163.57, 156.54, 152.66, 148.99, 143.45, 125.78, 124.66, 123.48, 59.79, 52.57, 34.91, 12.87, 10.39. HR-MS (ESI): calcd for C_19_H_17_F_5_N_5_O_2_, [M + H]^+^ 442.1302; found: 442.1307. Purity: 99.53%.

#### 2.3.8. N-((1-((6-chloropyridin-2-yl)methyl)-1H-1,2,3-triazol-4-yl)methyl)-2,3,4,5,6-pentafluorobenzamide (C7)

White solid, yield: 88%. Mp: 148–150 °C. ^1^H NMR (400 MHz, DMSO-d_6_) δ 9.44 (t, *J* = 5.3 Hz, 1H), 8.45 (d, *J* = 2.3 Hz, 1H), 8.14 (s, 1H), 7.81 (dd, *J* = 8.3, 2.5 Hz, 1H), 7.55 (d, *J* = 8.2 Hz, 1H), 5.69 (s, 2H), 4.54 (d, *J* = 5.5 Hz, 2H). ^13 ^C NMR (100 MHz, DMSO-d_6_) δ 156.55, 150.09, 149.47, 144.01, 139.55, 131.34, 124.39, 123.33, 49.38, 34.88. HR-MS (ESI): calcd for C_16_H_10_ClF_5_N_5_O, [M + H]^+^ 418.0494; found: 418.0497. Purity: 98.24%.

#### 2.3.9. N-((1-((1H-benzo[d]imidazol-2-yl)methyl)-1H-1,2,3-triazol-4-yl)methyl)-2,3,4,5,6-pentafluorobenzamide (C8)

White solid, yield: 73%. Mp: 232–234 °C. ^1^H NMR (400 MHz, DMSO-d_6_) δ 12.68 (s, 1H), 9.46 (t, *J* = 5.4 Hz, 1H), 8.13 (s, 1H), 7.55 (dd, *J* = 35.7, 7.6 Hz, 2H), 7.34 − 6.97 (m, 2H), 5.88 (s, 2H), 4.57 (d, *J* = 5.6 Hz, 2H). ^13 ^C NMR (100 MHz, DMSO-d_6_) δ 156.59, 148.24, 143.84, 123.66, 47.24, 34.89. HR-MS (ESI): calcd for C_18_H_12_F_5_N_6_O, [M + H]^+^ 423.0993; found: 423.0998. Purity: 98.15%.

### 2.4. *In vitro* enzymatic activity against FTO

Of 50 µL of buffer solution containing 2 mM L-ascorbic acid, 2 nM FTO, 2 M ssRNA, 280 µM (NH_4_)_2_Fe(SO_4_)_2_, 1 mM α-KG, and 50 mM Tris–HCl was prepared to perform the enzymatic reaction. Compounds with different concentrations were added into the solution and incubated at room temperature for 30 min. Then, the enzymatic reaction was quenched by heating at 95 °C for 5 min. ssRNA, nuclease P1, NH_4_OAc, NH_4_HCO_3_, and alkaline phosphatase were added and incubated at 37 °C for 3 h. Finally, the nucleosides were separated and detected using a Thermo TSQ Quantum Ultra LC/MS (Thermo Fisher Scientific, Waltham, America). Concentration-response curves were fitted with GraphPad Prism version 6.0 (GraphPad Software, San Diego, CA).

### 2.5. Cell proliferation assay

KYSE-150, KYSE-270, TE-1, KYSE-510, and EC109 cell lines were purchased from Shanghai Yuanye Biotechnology Co., LTD (Shanghai, China). EC109&shFTO and EC109&shControl cell lines were supported by Servicebio (Wuhan, China). All these cells were maintained in RPMI-1640 medium (Shanghai Yuanye Biotechnology Co., LTD, Shanghai, China) with 10% foetal bovine serum (Shanghai Yuanye Biotechnology Co., LTD, Shanghai, China) and 1% penicillin-streptomycin in a humidified atmosphere of 5% CO_2_ at 37 °C. Cells were cultured with compounds at different concentrations for 72 h. Next, 5 mg/mL MTT (Servicebio, Wuhan, China) was added and incubated for 4 h. DMSO was added into the system and shocked for 10 min. The absorbance at 490 nm was measured by using a multifunction microplate reader (Thermo Fisher Scientific, Waltham, MA).

### 2.6. Western blotting

RIPA buffer solution (Servicebio, Wuhan, China) was used to perform western blot. Of 20 µL of protein solution were subjected to SDS-PAGE, and then transferred to nitrocellulose membranes (Shanghai Yuanye Biotechnology Co., LTD, Shanghai, China). Of 5% non-fat milk (Servicebio, Wuhan, China) was used to block the system and nitrocellulose membranes were incubated at 4 °C with the first antibody overnight, followed by the incubation with a secondary antibody. Finally, blots were visualised by the chemiluminescence kit (Shanghai Yuanye Biotechnology Co., LTD, Shanghai, China).

### 2.7. Molecular docking studies

Studies of molecular modelling in this work were performed with Autodock software (The Scripps Research Institute, San Diego, CA). The crystal structure of FTO (PDB code: 5DAB) was downloaded from the RCSB protein database (http://www.rcsb.org/). Hydrogen-bond interaction between FTO and compounds was analysed by Pymol software (DeLano Scientific LLC, San Carlos, CA).

### 2.8. Cell cycle arrest

Cells were incubated with the targeted compound at different concentrations for 48 h and harvested. Of 70% cold ethanol was added and incubated at 4 °C for 12 h. Then, fixed cells were washed with PBS (Servicebio, Wuhan, China). Finally, the system was stained with PI (Servicebio, Wuhan, China) for 30 min under the dark condition and analysed by a Flow cytometer (Annoron, Beijing, China).

### 2.9. Xenograft study

BALB/c nude mice purchased from Shanghai Yuanye Biotechnology Co., LTD (Shanghai, China). All the animal experiments were performed according to approved guidelines from the ethics committee of Zhengzhou University (Approval number ZZU-2021–014). EC109 cell line was used to establish xenograft models in this work. Intragastric administration was adopted to finish the *in vivo* experiments. Organs (Heart, liver, spleen, lung, and kidney) from BALB/c mice for toxicity studies were fixed in 4% formaldehyde solution (Servicebio, Wuhan, China). Section of tissues was supported by Servicebio (Wuhan, China) and it was analysed by haematoxylin and eosin staining.

### 2.10. Statistical analysis

In order to maintain accuracy, each biological assay was repeated three times. In this work, **p < 0.01 was considered significant.

## 3. Results and discussion

### 3.1. Chemistry

**Scheme 1.**
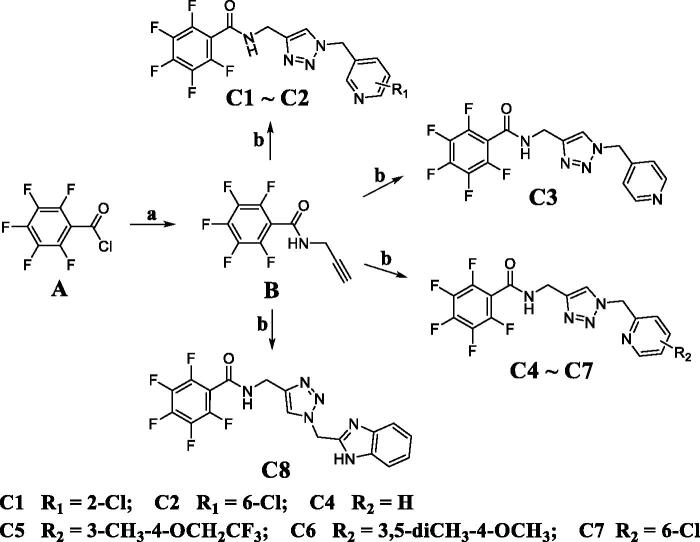
Reagents and conditions: (a) prop-2-yn-1-amine, triethylamine, dichloromethane; (b) Azide derivatives, CuSO_4_.5H_2_O, sodium ascorbate, and THF/H_2_O.

Synthetic route of 1,2,3-triazole-pyridine hybrids **C1**∼**C8** is displayed in Scheme 1. Intermediate **B** was obtained from the acylation reaction of prop-2-yn-1-amine with 2,3,4,5,6-pentafluorobenzoyl chloride in dichloromethane. Then, click reaction of intermediate **B** and appropriately substituted azide derivatives was performed to generate 1,2,3-triazole-pyridine hybrids **C1** ∼ **C8** in the mixed solvent system (tetrahydrofuran: water = 1: 1).

### 3.2. Antiproliferative activity of compound B and compound C1∼C8 against esophageal cancer

1,2,3-Triazole-pyridine hybrids **C1**∼**C8** were evaluated for their antiproliferative activities against five esophageal cancer cell lines (KYSE-150, KYSE-270, TE-1, KYSE-510, and EC109) by the MTT assay. 5-Fluorouracil (5-Fu) was employed as the reference drug in the reported reference for the antiproliferative evaluation of 1,2,3-triazole derivatives^15^. So, it also was used as the control to evaluate the accuracy of antiproliferative results. The antiproliferative results of compound **B** and 1,2,3-triazole-pyridine hybrids **C1**∼**C8** are summarised in Table 1.

**Table 1.** Antiproliferative activities against esophageal cancer cell lines

Compd.	IC_50_ (μM)				
	KYSE-150	KYSE-270	TE-1	KYSE-510	EC109
**B**	>20	>20	>20	>20	>20
**C1**	11.37 ± 0.69	12.26 ± 0.80	14.33 ± 0.68	13.91 ± 0.37	18.65 ± 0.92
**C2**	14.60 ± 0.16	9.34 ± 0.29	10.27 ± 0.91	9.34 ± 0.21	15.22 ± 0.36
**C3**	10.49 ± 0.38	14.16 ± 0.90	17.31 ± 0.68	11.07 ± 0.16	6.34 ± 0.11
**C4**	6.55 ± 0.20	7.83 ± 0.19	9.17 ± 0.09	8.12 ± 0.72	5.29 ± 0.28
**C5**	4.04 ± 0.16	2.04 ± 0.57	3.09 ± 0.10	5.07 ± 0.46	3.18 ± 0.27
**C6**	2.17 ± 0.59	1.35 ± 0.05	0.95 ± 0.07	4.15 ± 0.13	0.83 ± 0.04
**C7**	6.21 ± 0.14	5.29 ± 0.20	6.19 ± 0.25	9.22 ± 0.14	5.87 ± 0.27
**C8**	3.59 ± 0.08	2.04 ± 0.21	1.76 ± 0.09	5.13 ± 0.17	1.66 ± 0.04
**5-Fluorouracil**	17.39 ± 0.21	13.05 ± 0.62	7.73 ± 0.18	13.02 ± 0.70	9.62 ± 0.35

As shown in Table 1, compound **B** without the triazole-pyridine unit displayed the weak inhibitory activity against all esophageal cancer cell lines. However, 1,2,3-triazole-pyridine hybrids **C1**∼**C8** with the triazole-pyridine unit exhibited inhibitory activity against all esophageal cancer cell lines with IC_50_ values from 0.83 to 18.65 μM. These results indicated that triazole-pyridine unit might play the potential synergistic effects for inhibitory activity against esophageal cancer. Among all hybrids, compound **C6** displayed the best antiproliferative activity against KYSE-150, KYSE-270, TE-1, KYSE-510, and EC109 cell lines with IC_50_ values of 2.17, 1.35, 0.95, 4.15, and 0.83 μM, respectively.

In order to investigate the effects of substituent groups attaching to pyridine ring for the antiproliferative activity, 1,2,3-triazole-pyridine hybrids **C4**∼**C7** containing different substituent groups were synthesised and evaluated. Compound **C4** displayed moderate inhibitory activity against TE-1 cell line with an IC_50_ value of 9.17 μM. Replacing the hydrogen atom with a chlorine atom (**C7**, 6.19 μM) led to a small increment of activity against TE-1 cancer cells. However, changing the 3,5-dimethyl-4-methoxy group (**C6**) to a hydrogen atom (**C4**) or a chlorine atom (**C7**) led to a decrease of activity against KYSE-150, KYSE-270, TE-1, KYSE-510, and EC109 cells. All these results illustrated that substituent groups attaching to pyridine ring played the important role for the antiproliferative activity against esophageal cancer.

Furthermore, replacing the pyridine fragment of compound **C6** with 1*H*-benzo[*d*]imidazole of compound **C8** led to a decrease of activity against all esophageal cancer cell lines. Compound **C8** displayed the antiproliferative activity against KYSE-150, KYSE-270, TE-1, KYSE-510, and EC109 cell lines with IC_50_ values of 3.59, 2.04, 1.76, 5.13, and 1.66 μM, respectively. Therefore, 1,2,3-triazole-pyridine hybrid might be a promising scaffold to develop antitumor agents against esophageal cancer.

### 3.3. 1,2,3-Triazole-pyridine hybrids were novel FTO inhibitors

**Figure 2.**
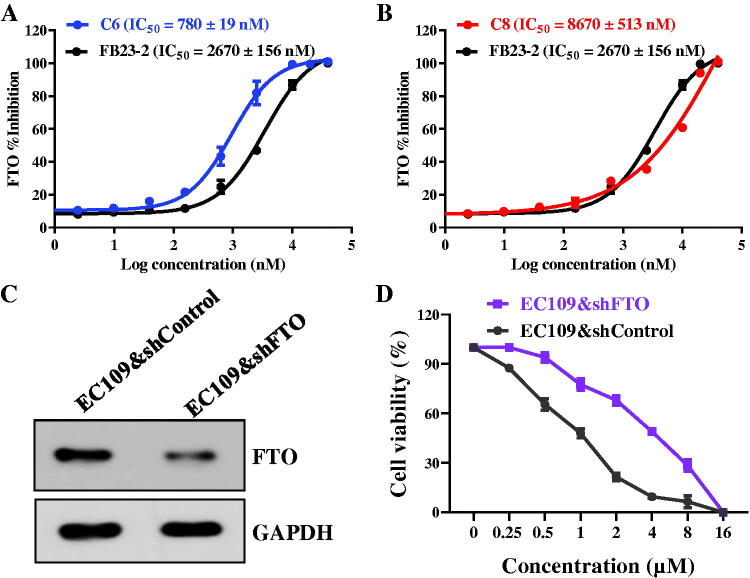
1,2,3-Triazole-pyridine hybrids were novel FTO inhibitors. (A) The inhibitory activity of compound **C6** against FTO; (B) The inhibitory activity of compound **C8** against FTO; (C) Expression of FTO in EC109&shFTO and EC109&shControl cells; (D) Cell viability of compound **C6** against EC109&shFTO and EC109&shControl cells. FB23-2 as a reported FTO inhibitor was the reference molecule.

Because of potent antiproliferative activity against all esophageal cancer cell lines, compound **C6** and **C8** were selected to evaluate their inhibitory activity against FTO. From the results of enzymatic inhibitory activity in Figure 2(A,B), compound **C6** and **C8,** respectively, displayed the potent inhibitory effects against FTO with IC_50_ values of 780 and 8670 nM. These results indicated that 1,2,3-Triazole-pyridine hybrids might be potential FTO inhibitors.

Recent references showed that FTO is aberrantly upregulated in various cancers, and down-regulation of LSD1 by RNAi or pharmacological inhibition has been an effective strategy to suppress the development of various cancer^16^. In this work, EC109&shFTO (FTO knock-down cells) and EC109&shControl (control cells) cell lines were cultured to investigate the *in vitro* antiproliferative activity of FTO inhibitors. Firstly, the expression of FTO in EC109&shFTO and EC109&shControl cells was detected and the results are shown in Figure 2(C). Then, MTT assay was performed to examine the antiproliferative activity of FTO inhibitor **C6**. From the results of Figure 2(D), FTO inhibitor **C6** exhibited inhibitory effects with an IC_50_ value of 1.06 μM against EC109&shControl cells. In contrast, compound **C6** inhibited EC109&shFTO cells with an IC_50_ value of 3.97 μM, about 3–4 fold less potent against EC109&shControl cells. The activity discrepancy in Figure 2(D) demonstrated that antiproliferative effects of compound **C6** against EC109 cells had a relationship with the FTO inhibition.

### 3.4. Molecular docking of FTO inhibitors

**Figure 3.**
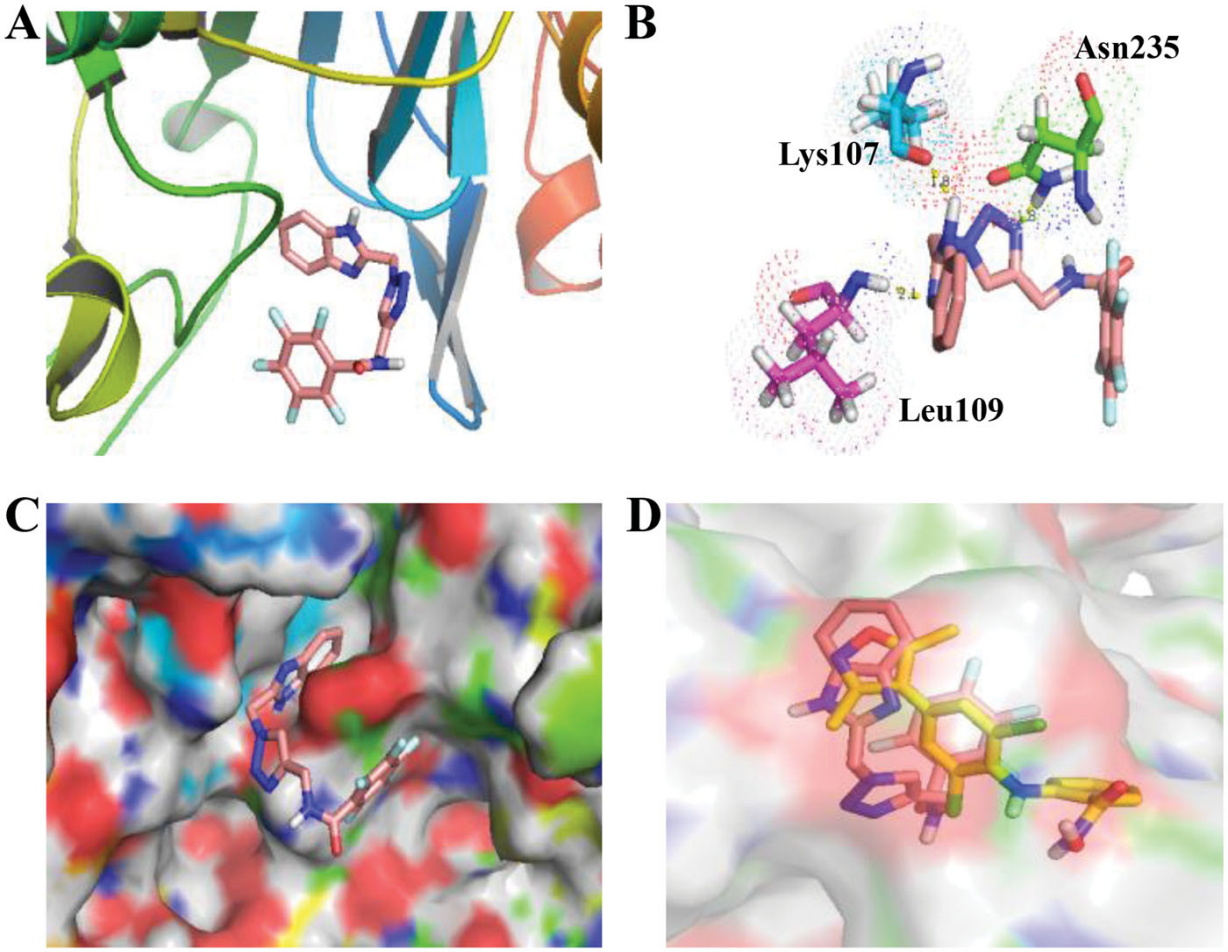
Molecular docking of compound **C8** (PDB code: 5DAB). (A) Compound **C8** binds to subunits of FTO; (B) Hydrogen-bond interaction of compound **C8** and FTO; (C) Compound **C8** locates into the active pocket of FTO; (D) a similar pocket between FB23-2 (yellow structure) and compound **C8**.

Due to different inhibitory effects of 1,2,3-Triazole-pyridine hybrids **C6** and **C8** against FTO, their molecular docking studies were investigated in this work. To predict the binding models between compounds and FTO, docking analysis using the Autodock software was performed. PDB code was 5DAB and FTO protein was downloaded from the RCSB protein database. As shown in Figure 3, compound **C8** locates into the active pocket of FTO and forms the hydrogen-bond interaction with Lys107, Leu109, and Asn235. As a reference molecule, FB23-2 was also docked using the same methods. From the results in Figure 3(D), FB23-2 as a reported FTO inhibitor (yellow structure) was docked into a similar pocket as compound **C8**.

**Figure 4.**
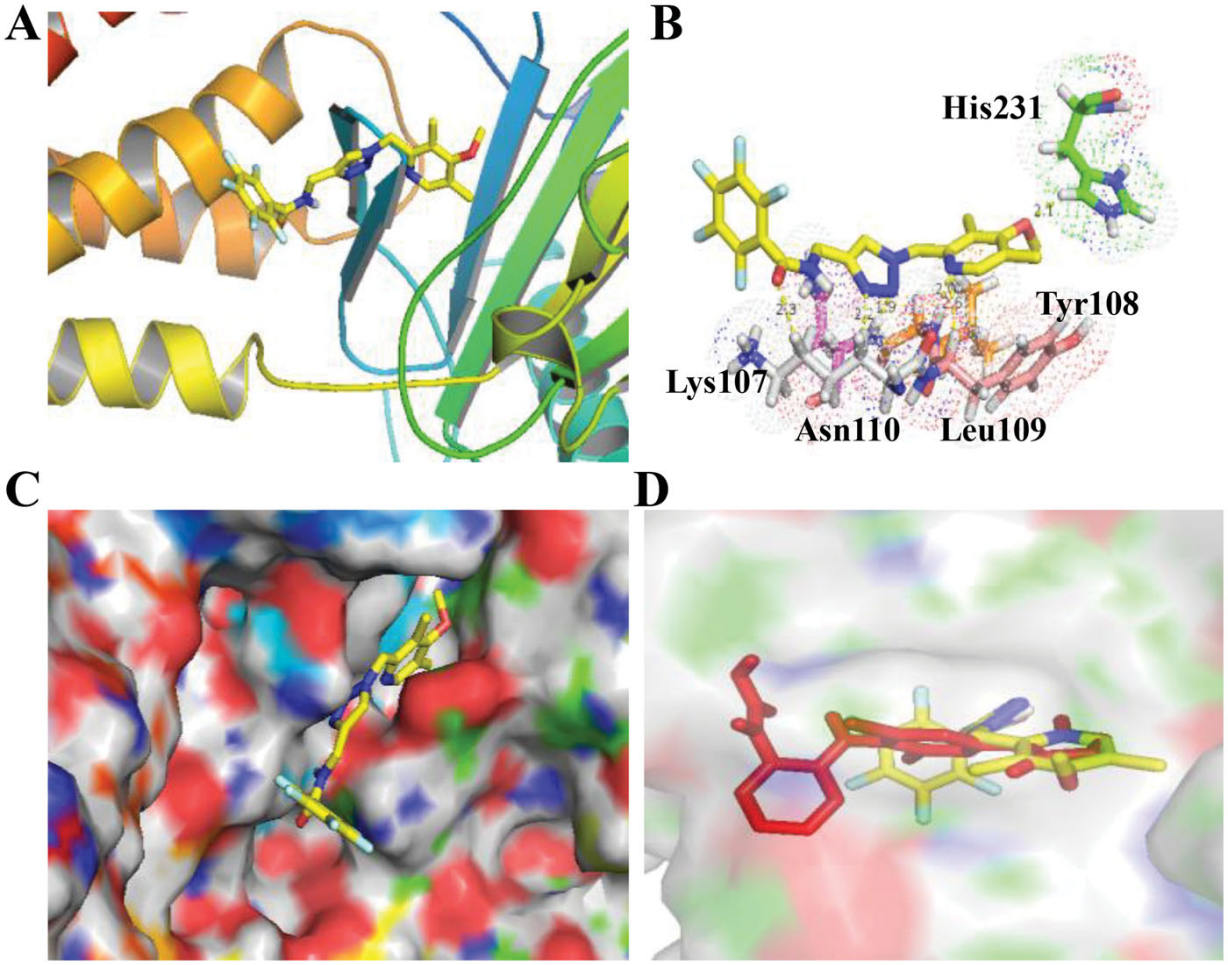
Molecular docking of compound **C6** (PDB code: 5DAB). (A) Compound **C6** binds to subunits of FTO; (B) Hydrogen-bond interaction of compound **C6** and FTO; (C) Compound **C6** locates into the active pocket of FTO; (D) a similar pocket between FB23-2 (red structure) and compound **C8**.

As shown in Figure 4, compound **C6** was docked into the active site of FTO and displayed the potent inhibitory activity against FTO. The amide group attaching to 1,2,3,4,5-pentafluorobenzene ring of 1,2,3-Triazole-pyridine hybrid **C6** formed a hydrogen bond with Lys107. 1,2,3-Triazole unit of compound **C6** formed two hydrogen bonds with Lys107 and Asn110 of FTO. In addition, pyridine unit of compound **C6** also formed two hydrogen bonds with Tyr108 and Leu109 of FTO. From the results in Figure 4(D), FB23-2 as a reported FTO inhibitor (red structure) was docked into a similar pocket as compound **C6**.

### 3.5. Cell cycle analysis

**Figure 5.**
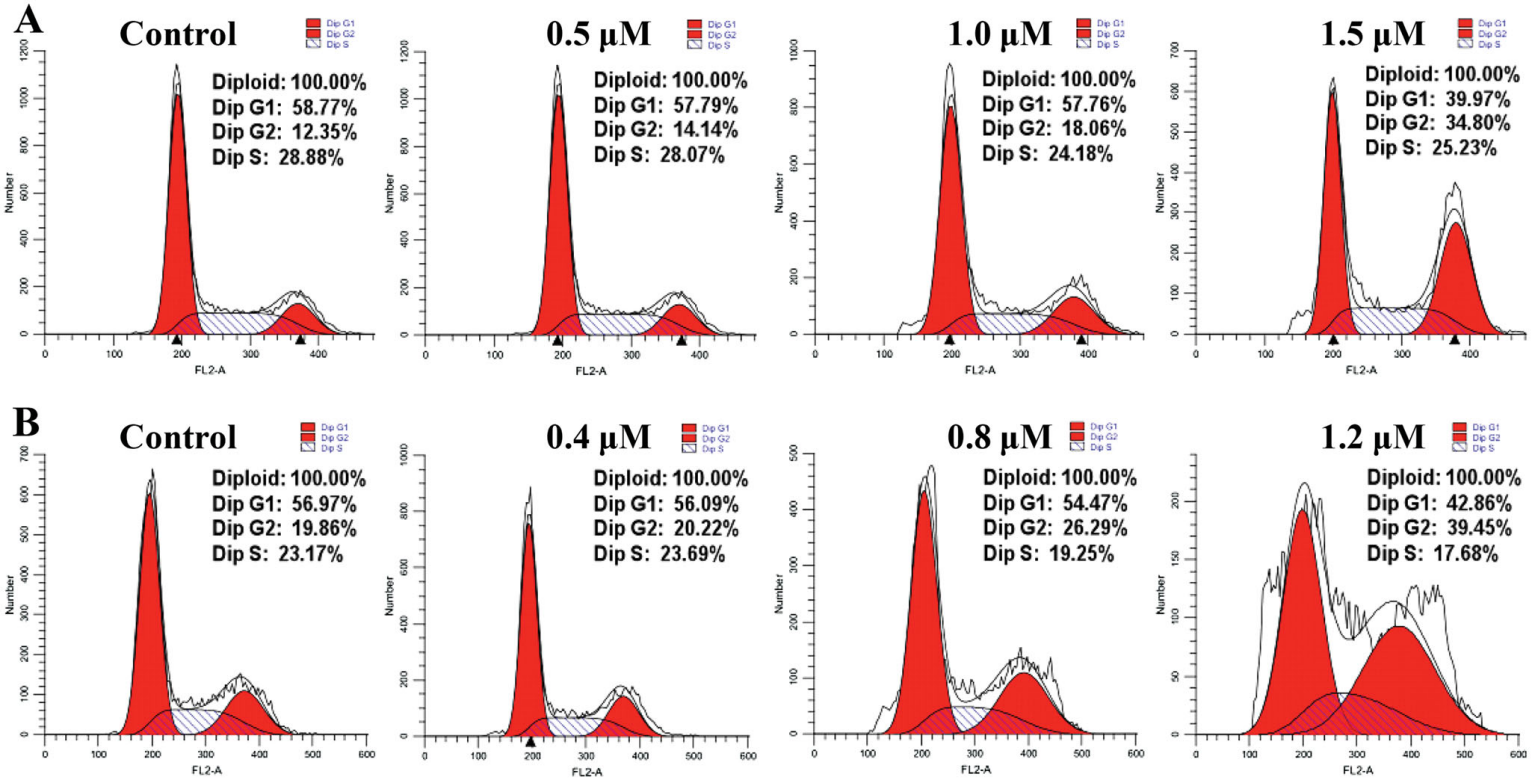
Cell cycle analysis of compound **C6** against TE-1 (A) and EC109 (B) cells.

*N*^6^-Methyladenosine (m^6^A) modification is the major chemical modification in mRNA that controls cell proliferation and cell cycle^17^. Recent studies reported that FTO demethylates *Cyclin D1* mRNA and controls cell cycle progression^18^. In order to explore the effects of cell cycle of compound **C6**, TE-1 and EC109 cell lines were selected according to its antiproliferative activity results. From the cell cycle analysis of compound **C6** at different concentrations against TE-1 and EC109 cells in Figure 5(A), it arrested cell cycle at G2 phase accompanying with the decrease of cells at G1 and S phase in a concentration-dependent manner. These findings indicated that compound **C6 as** a novel FTO inhibitor could arrest cell cycle against esophageal cancer.

### 3.6. The regulation of epithelial mesenchymal transition (EMT) pathway

**Figure 6.**
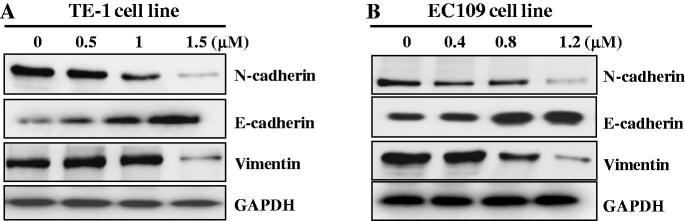
Compound **C6** regulated EMT pathway against TE-1 (A) and EC109 (B) cells.

Epithelial mesenchymal transition (EMT) as a critical cellular programme in which epithelial cells undergo series of biochemical changes to acquire mesenchymal phenotype displays important roles in the development of esophageal cancer^19^. EMT is characterised by the upregulation of N-cadherin and the downregulation of E-cadherin, which is regulated by a complex network of signalling pathways and transcription factors^20^. To further explore the anticancer mechanism of FTO inhibitor **C6**, TE-1, and EC109 cell lines were treated for 48 h. From the results in Figure 6, the expression levels of N-cadherin and Vimentin were decreased and the expression level of E-cadherin was increased against TE-1 and EC109 cells in a concentration-dependent manner, demonstrating that FTO inhibitor **C6** could inhibit EMT pathway against esophageal cancer.

### 3.7. The regulation of PI3K/AKT pathway

**Figure 7.**
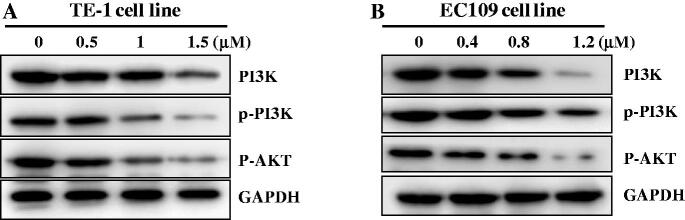
Compound **C6** regulated PI3K/AKT pathway against TE-1 (A) and EC109 (B) cells.

Dysregulation of FTO was implicated in multiple biological processes including proliferation and cell cycle against different tumors^21^. Importantly, these modulations might rely on the communications between FTO and PI3K/AKT signalling pathway^22^. In recent years, studies have shown that components of the PI3K/Akt signalling pathway are frequently altered in esophageal cancer^23^. TE-1 and EC109 cell lines were treated with FTO inhibitor **C6** at different concentrations for 48 h to perform western blot. From the results in Figure 7, the expression levels of PI3K, p-PI3K, and p-AKT were decreased against TE-1 and EC109 cells in a concentration-dependent manner, indicating that FTO inhibitor C6 could regulate PI3K/AKT pathway against esophageal cancer.

### 3.8. *In vivo* antitumor study

**Figure 8.**
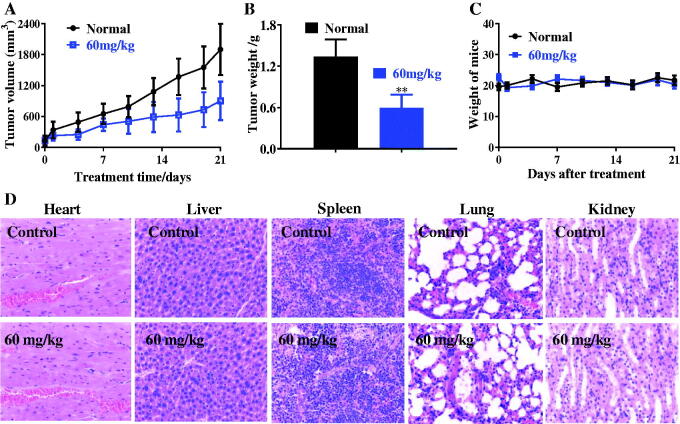
*In vivo* antitumor effects of compound **C6**. (A) Tumour volume; (B) Tumour weight; (C) Weight of mice; (D) Haematoxylin and eosin staining. **p < 0.01.

Due to the potent inhibitory activity of FTO inhibitor **C6** against esophageal cancer EC109 cell line, we also evaluated the *in vivo* anticancer effects of FTO inhibitor **C6** on xenograft models bearing EC109 cells. After the treatment of FTO inhibitor **C6** with the dose of 60 mg/kg, the tumour weight, the weight of mice and tumour volume were measured and recorded. From the results in Figure 8(A,B), FTO inhibitor **C6** inhibited tumour growth in esophageal cancer xenograft models remarkably. Weight of mice was almost unchanged in Figure 8(C) demonstrating that FTO inhibitor **C6** displayed the potent anticancer effects without obvious toxicities. In addition, main organs (Heart, liver, spleen, lung, and kidney) of oesophageal cancer xenograft models were performed haematoxylin and eosin staining. As shown in Figure 8(D), these results also suggested low global toxicities.

## 4. Conclusions

In summary, a novel series of 1,2,3-triazole-pyridine derivatives were synthesised according to click reaction and further evaluated for their inhibitory activity against five esophageal cancer cell lines (KYSE-150, KYSE-270, TE-1, KYSE-510, and EC109). Among them, compound **C6** displayed the most potent antiproliferative activity against KYSE-150, KYSE-270, TE-1, KYSE-510, and EC109 cell lines with IC_50_ value of 2.17, 1.35, 0.95, 4.15, and 0.83 μM, respectively. In this work, compound **C6** was identified as a novel FTO inhibitor and regulated EMT pathway and PI3K/AKT pathway against esophageal cancer. Importantly, *In vivo* antitumor effects of compound **C6** showed that it was an orally anticancer agent with potent effects and low toxicities. Therefore, FTO might be a potential therapeutic target in esophageal cancer, and compound **C6** could be a promising candidate for the drug discovery to treat esophageal cancer.

## Supplementary Material

Supplemental Material
