# Supplementary material for: Discovery of novel mRNA demethylase FTO inhibitors against esophageal cancer
Source: J Enzyme Inhib Med Chem. 2022 Jul 14;37(1):1995–2003. doi: 10.1080/14756366.2022.2098954 (PMC9291647; doi:10.1080/14756366.2022.2098954)
Supplement: Supplemental Material [file IENZ_A_2098954_SM6791.pdf]

Discovery of novel mRNA demethylase FTO inhibitors against esophageal cancer

Bo Qin[a#*], Qian Bai[b#], Dan Yan[a], Fanxiang Yin[a], Zhu Zhu[a], Chaoyuan Xia[a], Yang Yang[a*], Yi Zhao[a*]

[a]The First Affiliated Hospital of Zhengzhou University, No.1 Eastern Jianshe Road, Zhengzhou, Henan, China
[b]The Second Affiliated Hospital of Zhengzhou University, No.2 Jingba Road, Zhengzhou, Henan, China

[#]These authors contributed equally to this work.
[*]Corresponding author: Bo Qin (boqin@mail.ustc.edu.cn), Yang Yang (yangyangbio@163.com) and Yi Zhao (zhaoyi0910@163.com).

2,3,4,5,6-Pentafluoro-N-(prop-2-yn-1-yl)benzamide (B)

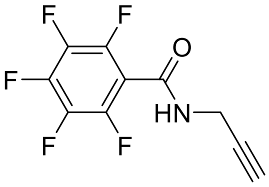

White solid, yield: 79%. Mp: 83-85 °C. ^{1}H NMR (400 MHz, DMSO-d_6) δ 9.40 (s, 1H), 4.10 (dd, J = 5.5, 2.5 Hz, 2H), 3.21 (s, 1H). ^{13}C NMR (100 MHz, DMSO-d_6) δ 156.41, 144.34, 141.87, 139.98, 138.16, 135.66, 111.95, 79.78, 73.67, 28.64. HR-MS (ESI): calcd for $C_{10}H_5F_5NO$, [M+H]$^+$ 250.0291; found: 250.0297. Purity: 96.82%.

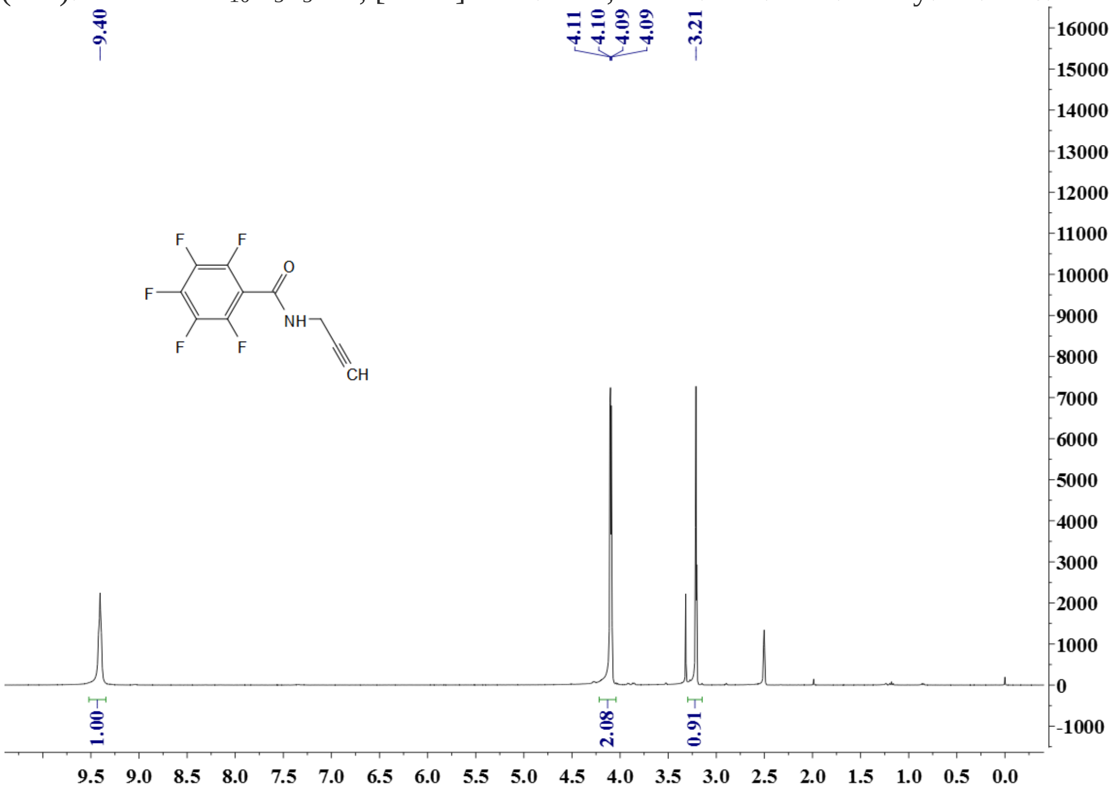

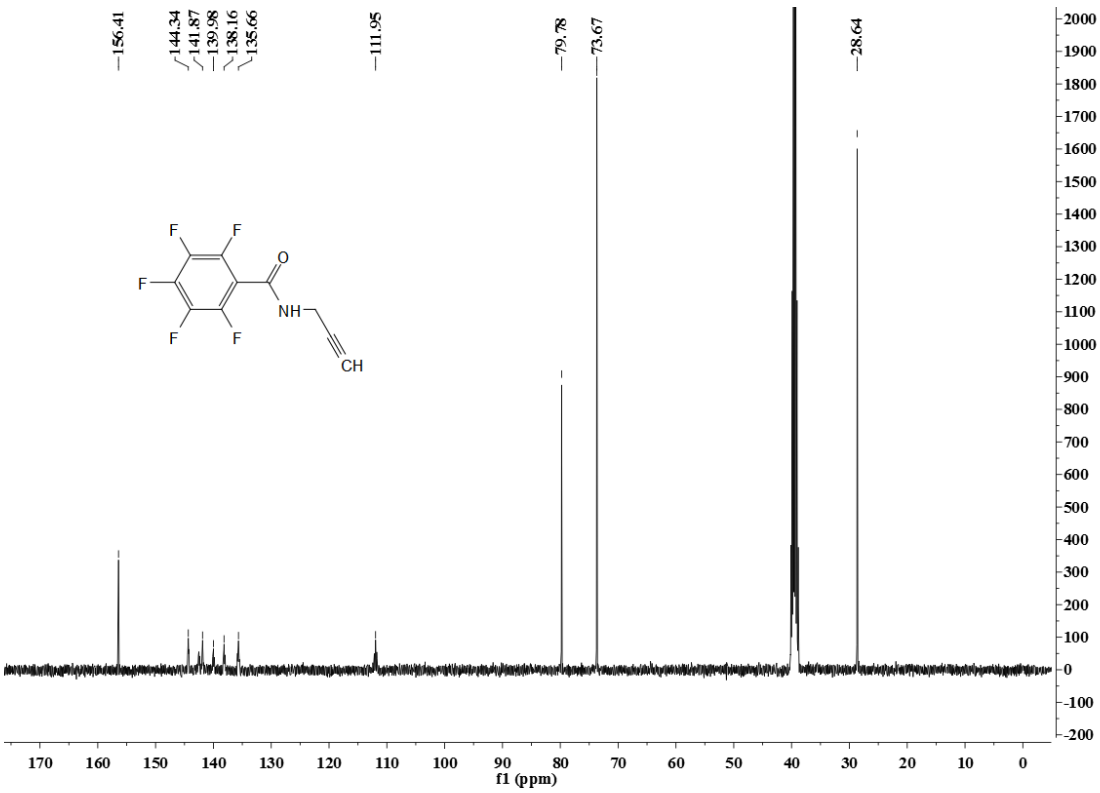

N-((1-((2-chloropyridin-3-yl)methyl)-1H-1,2,3-triazol-4-yl)methyl)-2,3,4,5,6-pentafluorobenzamide (C1)

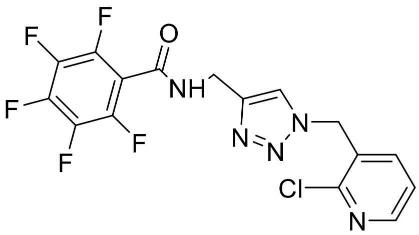

White solid, yield: 83%. Mp: 152-154 °C. ^{1}H NMR (400 MHz, DMSO-d_6) δ 9.44 (t, J = 5.4 Hz, 1H), 8.45 (d, J = 2.3 Hz, 1H), 8.13 (s, 1H), 7.81 (dd, J = 8.3, 2.5 Hz, 1H), 7.55 (d, J = 8.2 Hz, 1H), 5.69 (s, 2H), 4.53 (d, J = 5.6 Hz, 2H). ^{13}C NMR (100 MHz, DMSO-d_6) δ 156.55, 150.08, 149.48, 144.01, 139.57, 131.36, 124.41, 123.34, 49.37, 34.87. HR-MS (ESI): calcd for $C_{16}H_{10}ClF_5N_5O$, $[M+H]^+$ 418.0494; found: 418.0498. Purity: 97.43%.

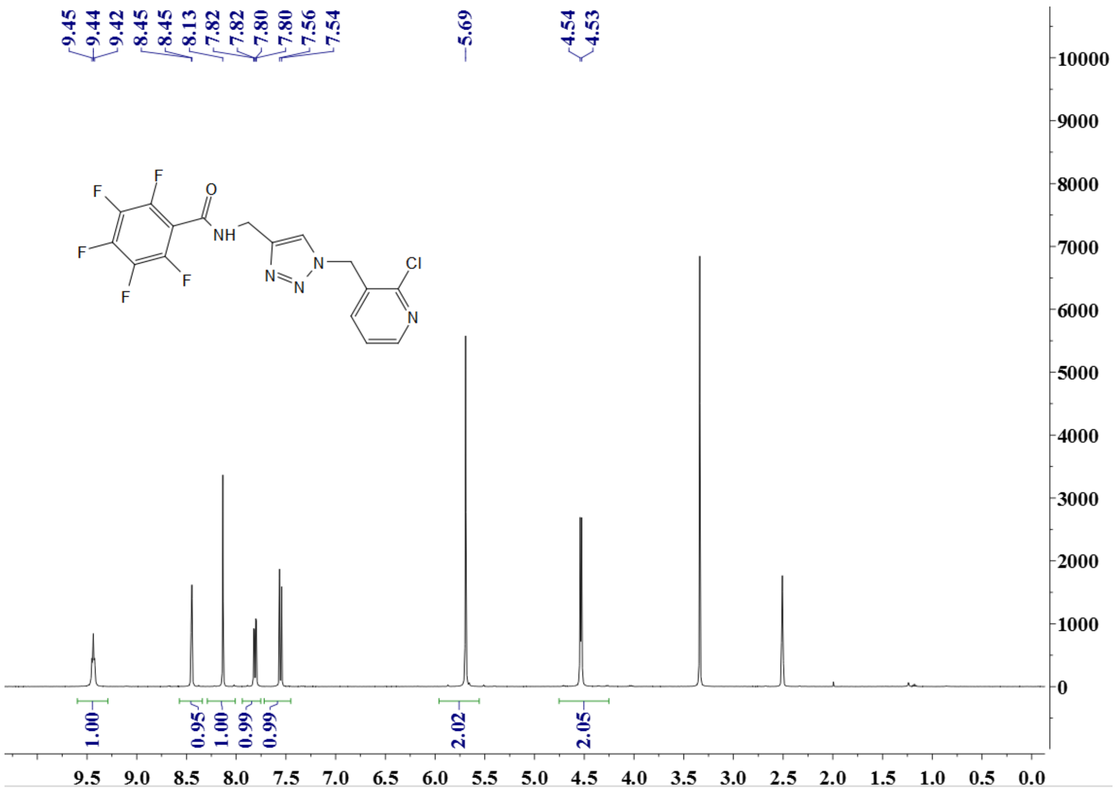

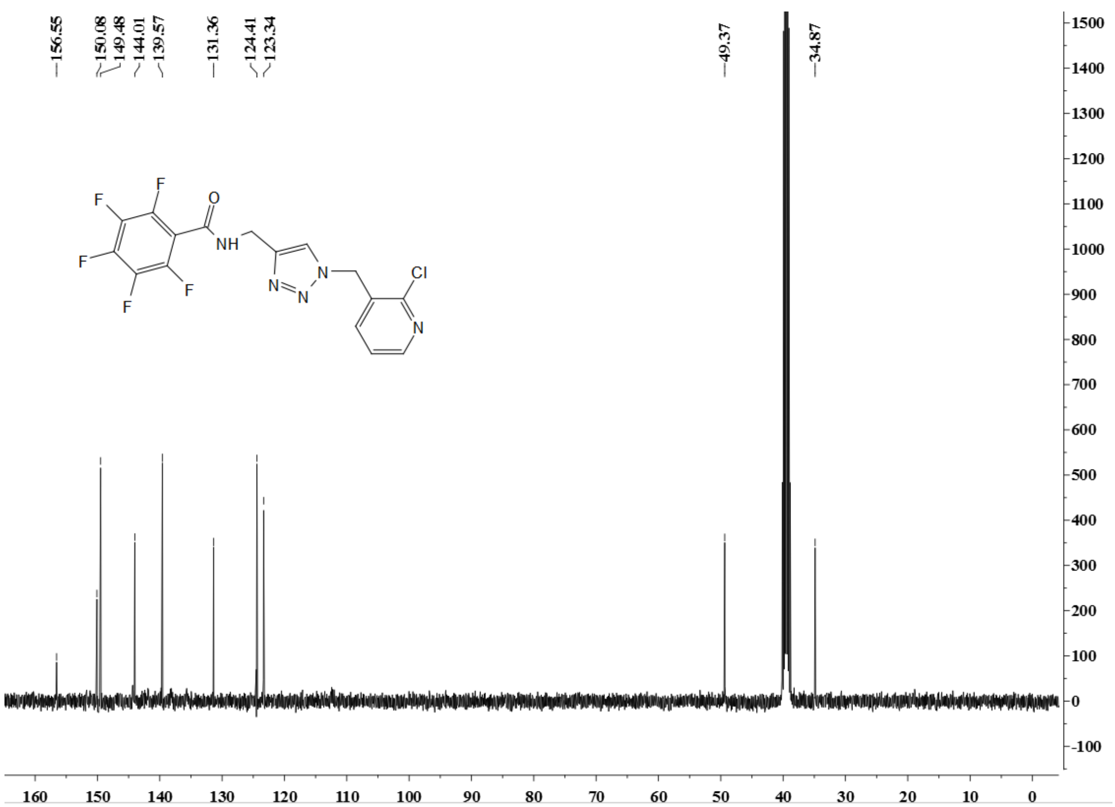

N-((1-((6-chloropyridin-3-yl)methyl)-1H-1,2,3-triazol-4-yl)methyl)-2,3,4,5,6-pentafluorobenzamide (C2)

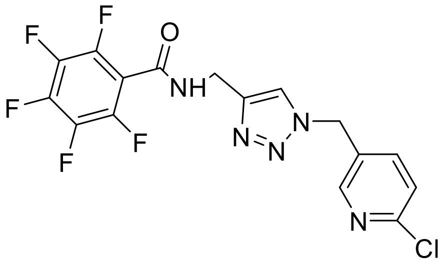

White solid, yield: 80%. Mp: 148-150 °C. ^{1}H NMR (400 MHz, DMSO-d_6) δ 9.44 (t, *J* = 5.4 Hz, 1H), 8.45 (d, *J* = 2.3 Hz, 1H), 8.14 (s, 1H), 7.81 (dd, *J* = 8.3, 2.5 Hz, 1H), 7.55 (d, *J* = 8.2 Hz, 1H), 5.69 (s, 2H), 4.53 (d, *J* = 5.6 Hz, 2H). ^{13}C NMR (100 MHz, DMSO-d_6) δ 156.55, 150.08, 149.48, 144.01, 139.57, 131.35, 124.40, 123.33, 49.37, 34.87. HR-MS (ESI): calcd for $C_{16}H_{10}ClF_5N_5O$, $[M+H]^+$ 418.0494; found: 418.0498. Purity: 97.57%.

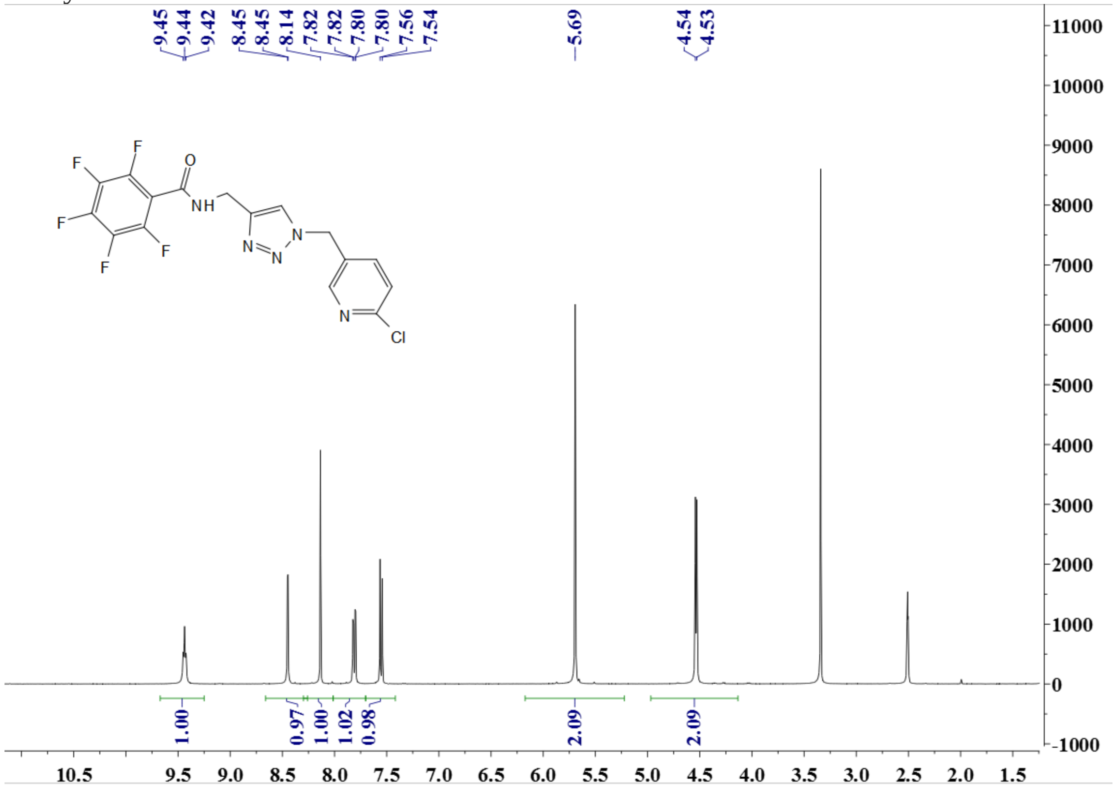

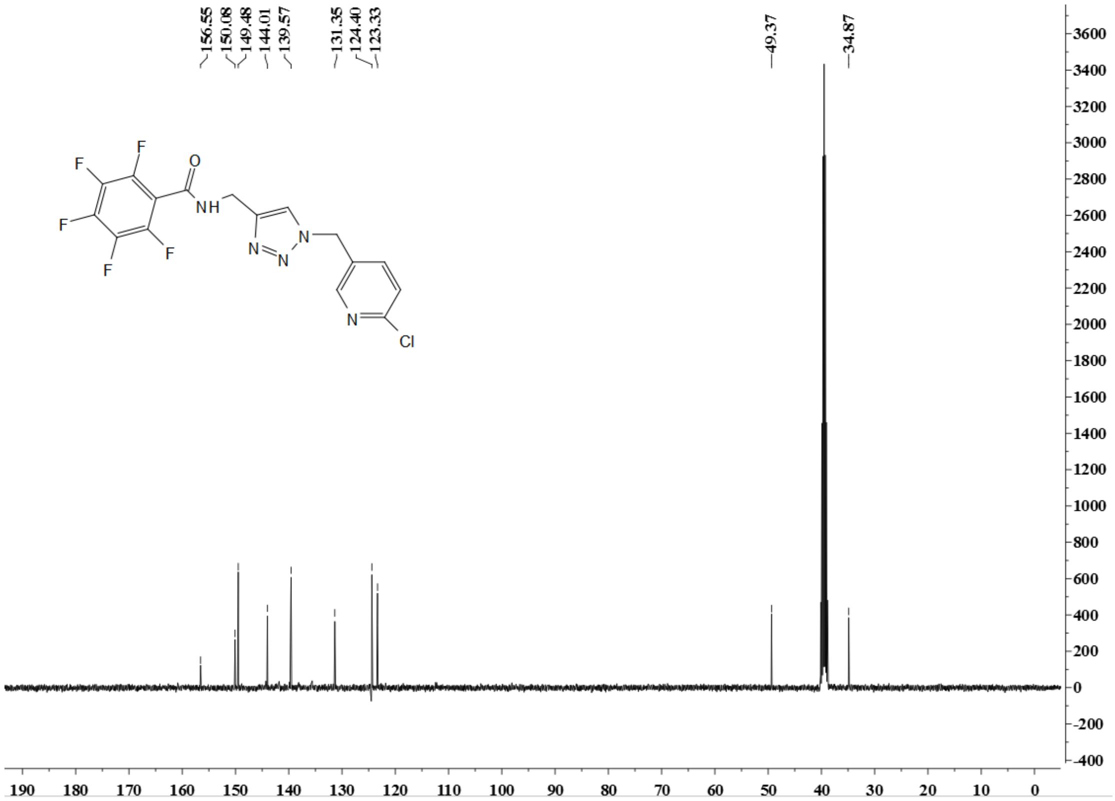

2,3,4,5,6-pentafluoro-N-((1-(pyridin-4-ylmethyl)-1H-1,2,3-triazol-4-yl)methyl)benzamide (C3)

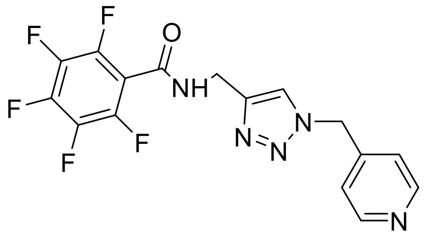

White solid, yield: 72%. Mp: 108-110 °C. ^{1}H NMR (400 MHz, DMSO-d_6) δ 9.49 (t, J = 5.4 Hz, 1H), 8.57 (d, J = 3.8 Hz, 2H), 8.16 (s, 1H), 7.21 (d, J = 5.6 Hz, 2H), 5.72 (s, 2H), 4.58 (d, J = 5.6 Hz, 2H). ^{13}C NMR (100 MHz, DMSO-d_6) δ 156.58, 149.96, 144.90, 144.02, 123.76, 122.24, 51.44, 34.89. HR-MS (ESI): calcd for $C_{16}H_{11}F_5N_5O$, $[M+H]^+$ 384.0884; found: 384.0889. Purity: 98.06%.

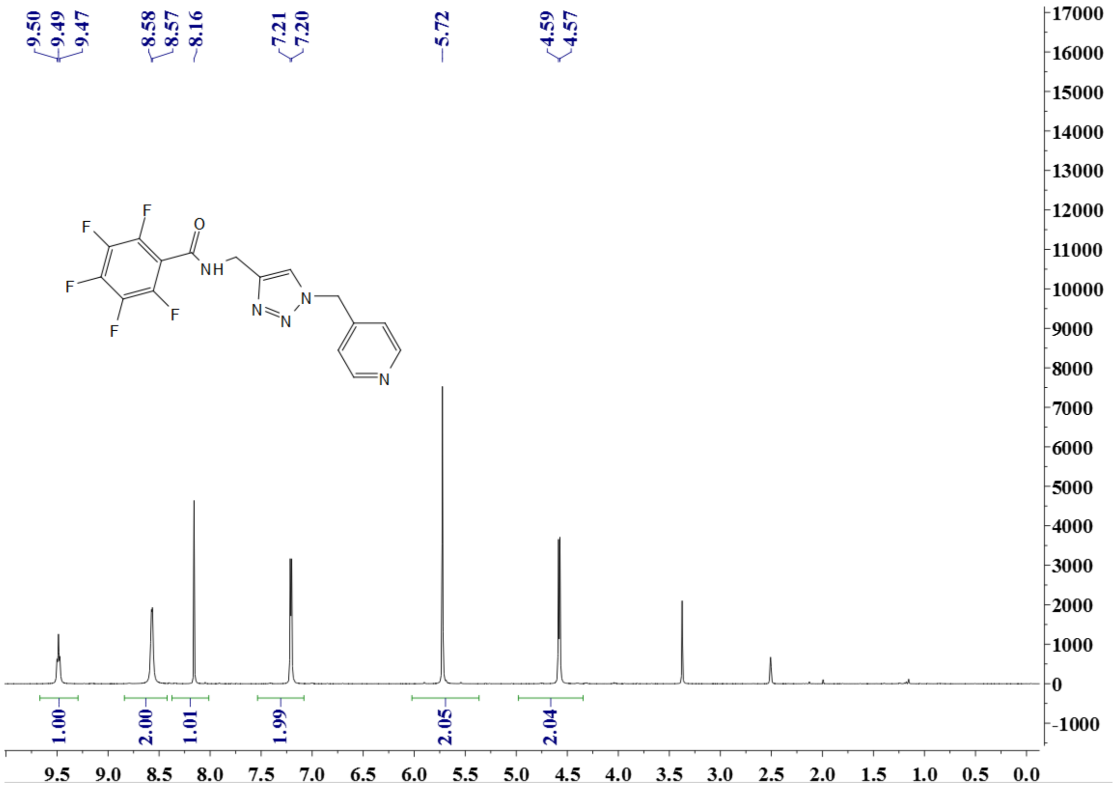

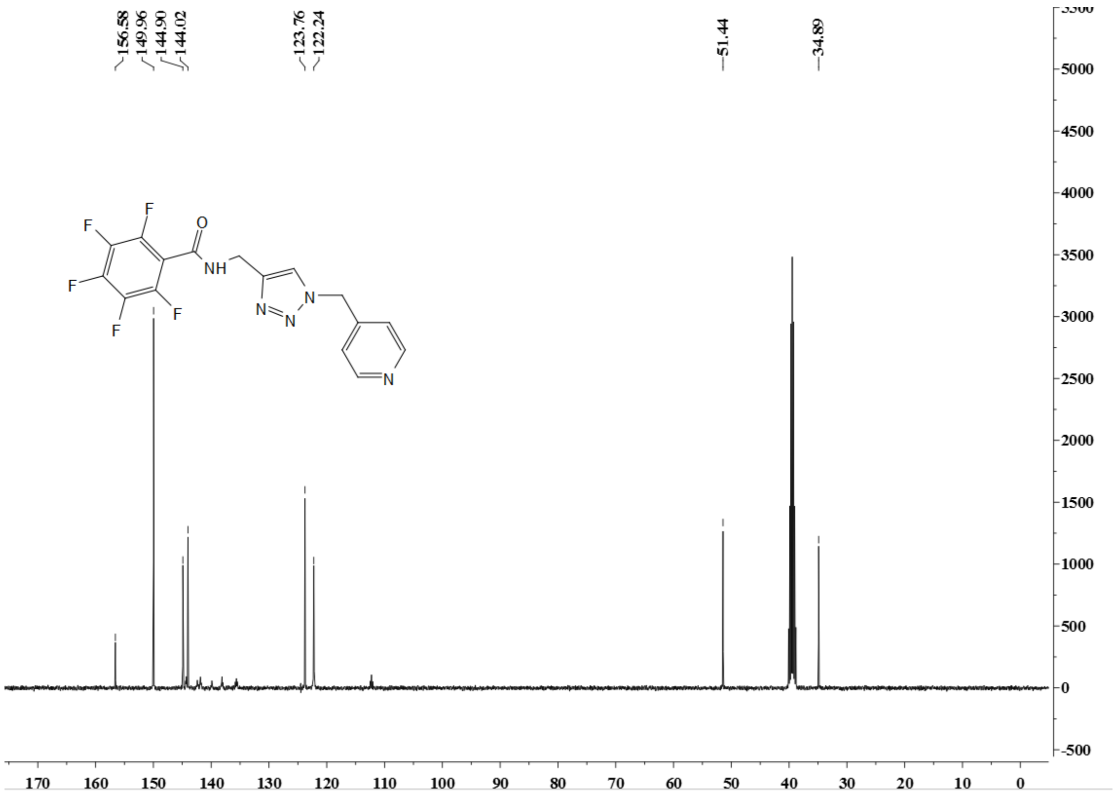

2,3,4,5,6-pentafluoro-N-((1-(pyridin-2-ylmethyl)-1H-1,2,3-triazol-4-yl)methyl)benzamide (C4)

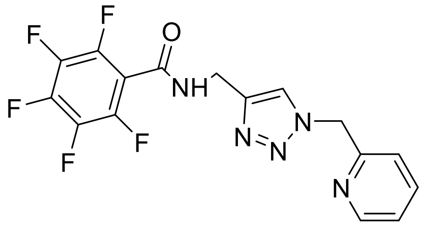

White solid, yield: 88%. Mp: 149-151 °C. ^{1}H NMR (400 MHz, DMSO-d_6) δ 9.47 (t, J = 5.3 Hz, 1H), 8.55 (d, J = 4.2 Hz, 1H), 8.08 (s, 1H), 7.83 (td, J = 7.7, 1.7 Hz, 1H), 7.60 – 7.09 (m, 2H), 5.73 (s, 2H), 4.56 (d, J = 5.6 Hz, 2H). ^{13}C NMR (100 MHz, DMSO-d_6) δ 156.55, 155.02, 149.40, 143.68, 137.27, 123.78, 123.20, 122.14, 54.34, 34.90. HR-MS (ESI): calcd for $C_{16}H_{11}F_5N_5O$, $[M+H]^+$ 384.0884; found: 384.0887. Purity: 96.99%.

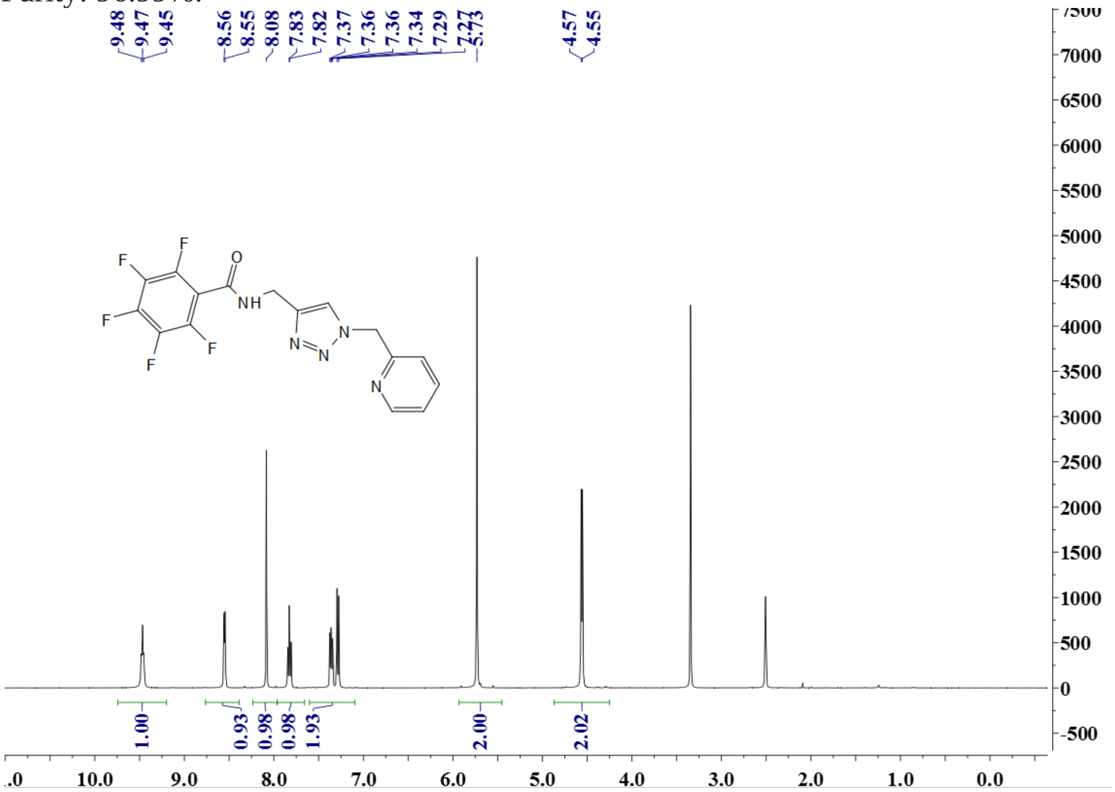

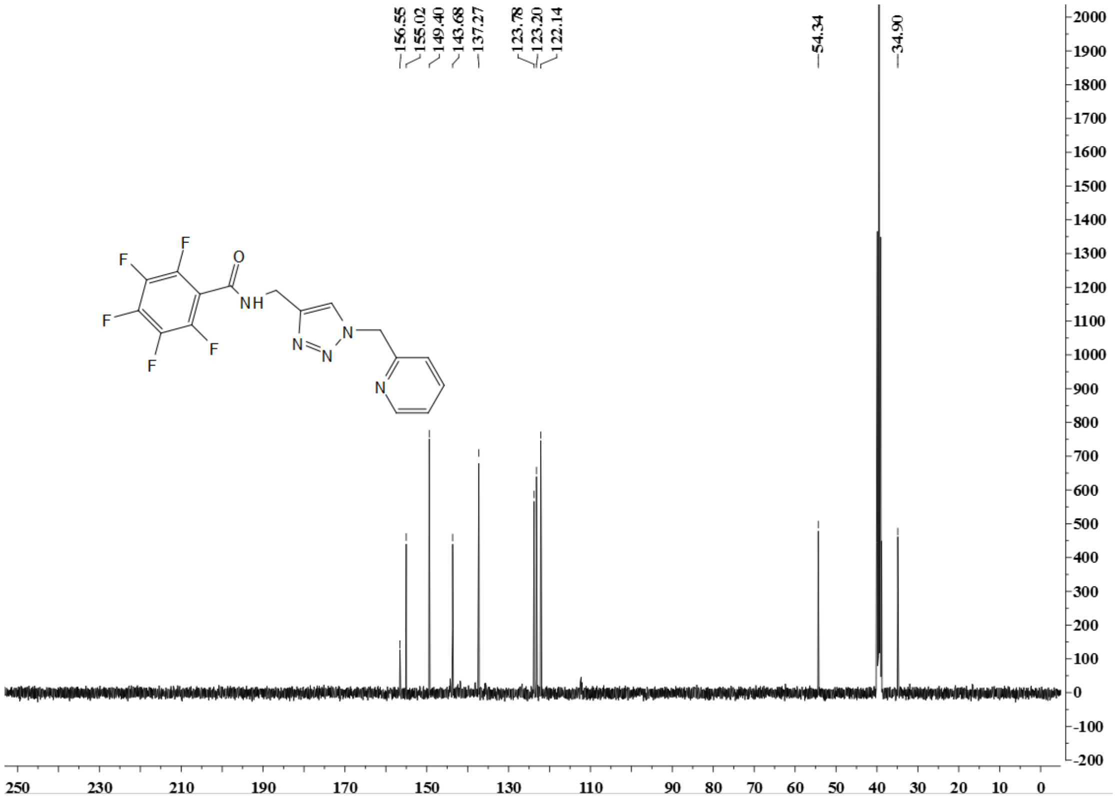

2,3,4,5,6-pentafluoro-N-((1-((3-methyl-4-(2,2,2-trifluoroethoxy)pyridin-2-yl)methyl)-1H-1,2,3-triazol-4-yl)methyl)benzamide (C5)

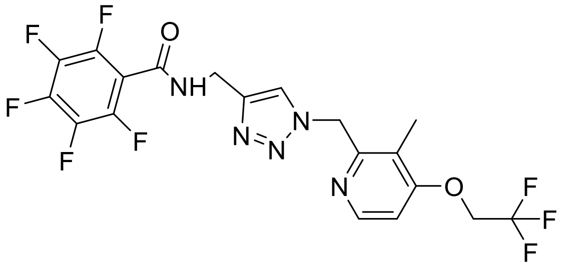

White solid, yield: 69%. Mp: 156-158 °C. ^{1}H NMR (400 MHz, DMSO-d_6) δ 9.44 (t, J = 5.5 Hz, 1H), 8.30 (d, J = 5.6 Hz, 1H), 7.92 (s, 1H), 7.13 (d, J = 5.7 Hz, 1H), 5.74 (s, 2H), 4.92 (q, J = 8.7 Hz, 2H), 4.54 (d, J = 5.6 Hz, 2H), 2.22 (s, 3H). ^{13}C NMR (100 MHz, DMSO-d_6) δ 161.30, 156.55, 153.95, 148.03, 143.45, 123.62, 120.06, 107.33, 64.82, 64.48, 52.43, 34.90, 9.70. HR-MS (ESI): calcd for $C_{19}H_{14}F_8N_5O_2$, $[M+H]^+$ 496.1020; found: 496.1025. Purity: 97.43%.

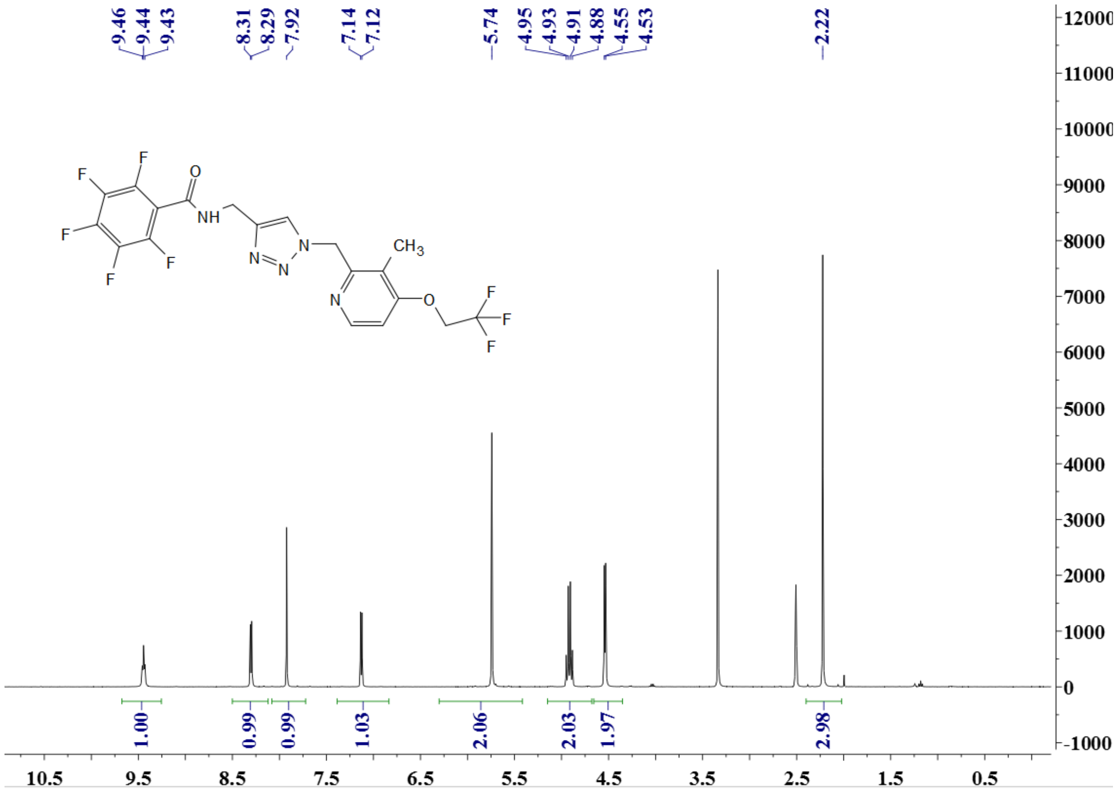

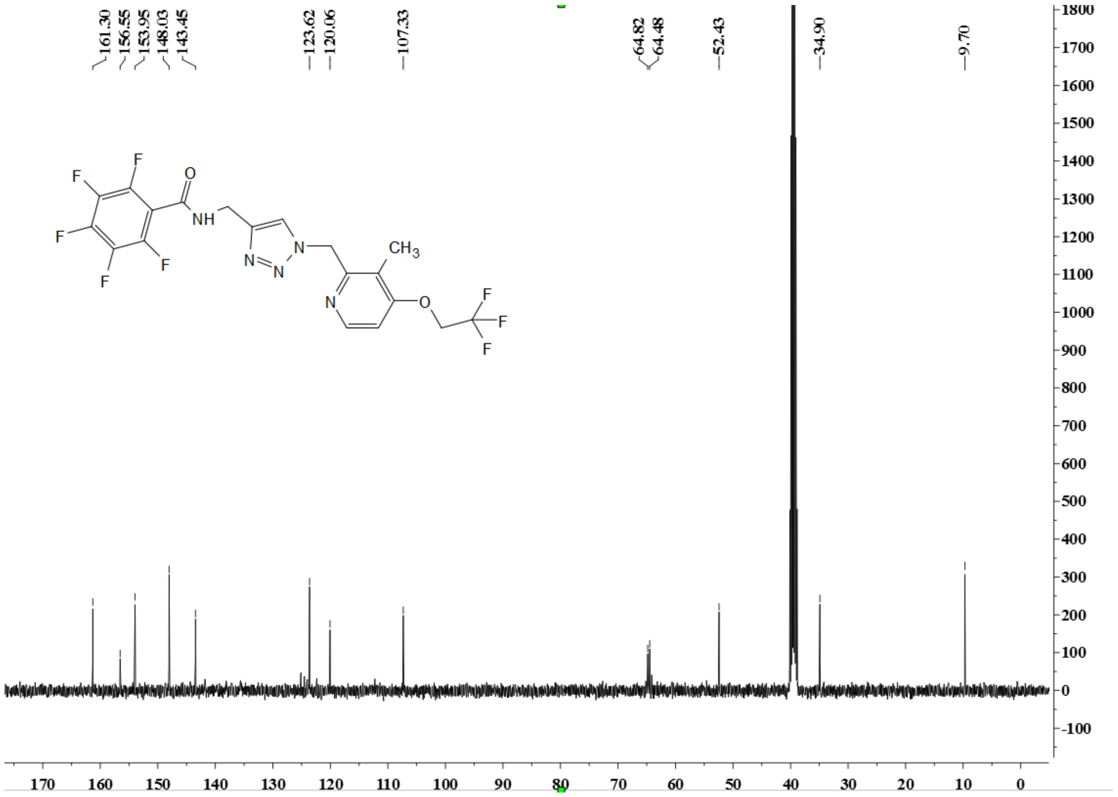

2,3,4,5,6-pentafluoro-N-((1-((4-methoxy-3,5-dimethylpyridin-2-yl)methyl)-1H-1,2,3-triazol-4-yl)methyl)benzamide (C6)

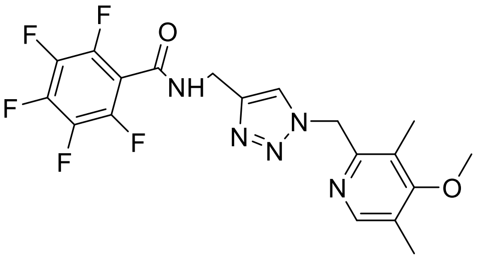

White solid, yield: 62%. Mp: 135-137 °C. ^{1}H NMR (400 MHz, DMSO-d_6) δ 9.44 (t, J = 5.3 Hz, 1H), 8.18 (s, 1H), 7.90 (s, 1H), 5.69 (s, 2H), 4.53 (d, J = 5.6 Hz, 2H), 3.73 (s, 3H), 2.23 (d, J = 19.3 Hz, 6H). ^{13}C NMR (100 MHz, DMSO-d_6) δ 163.57, 156.54, 152.66, 148.99, 143.45, 125.78, 124.66, 123.48, 59.79, 52.57, 34.91, 12.87, 10.39. HR-MS (ESI): calcd for $C_{19}H_{17}F_5N_5O_2$, $[M+H]^+$ 442.1302; found: 442.1307. Purity: 99.53%.

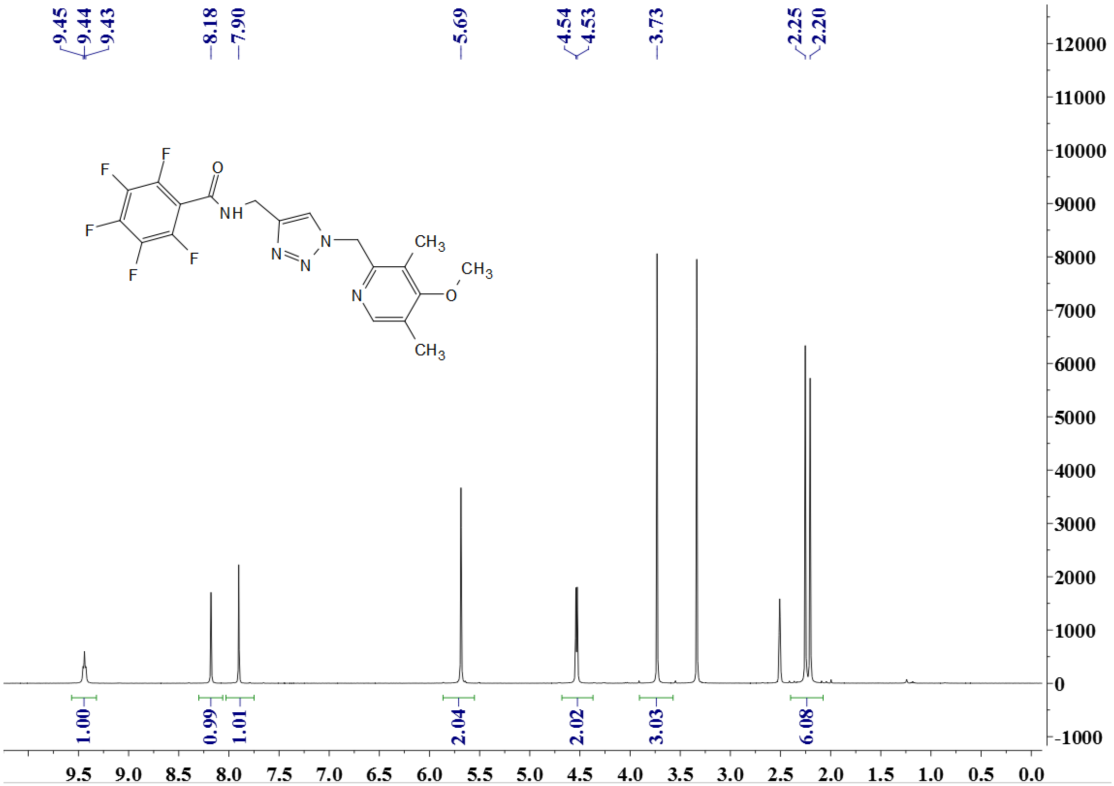

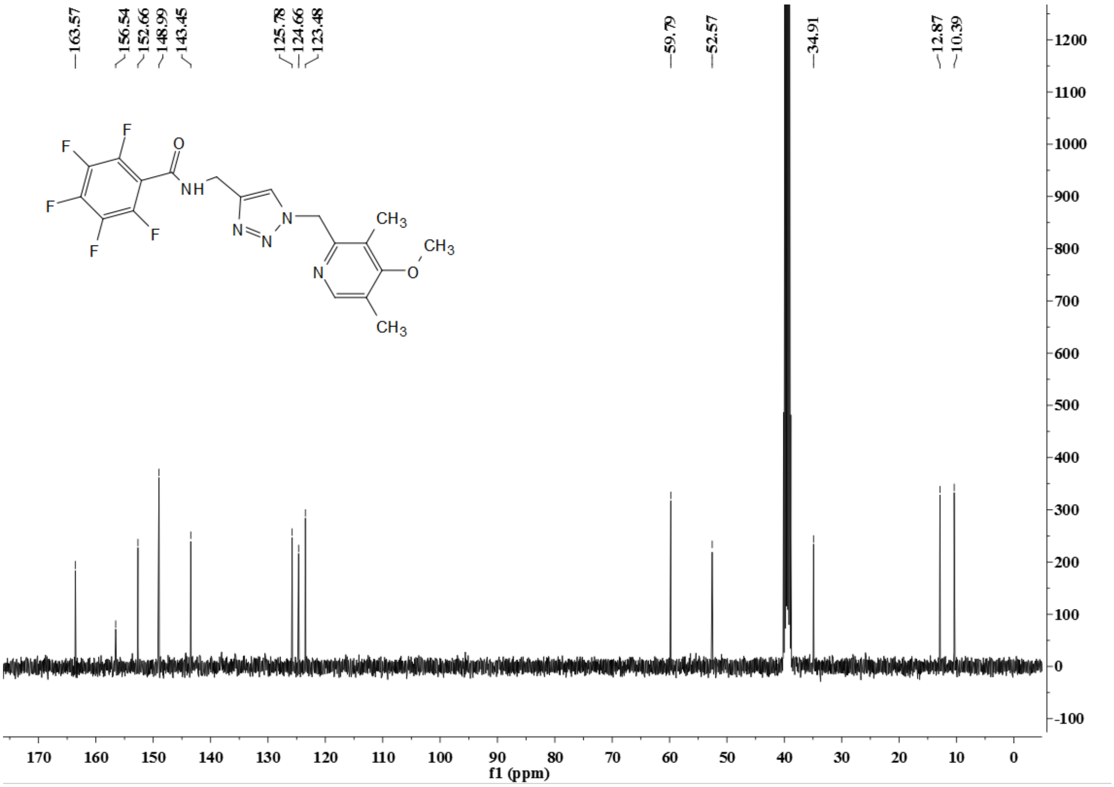

N-((1-((6-chloropyridin-2-yl)methyl)-1H-1,2,3-triazol-4-yl)methyl)-2,3,4,5,6-pentafluorobenzamide (C7)

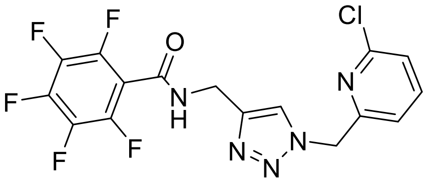

White solid, yield: 88%. Mp: 148-150 °C. ^{1}H NMR (400 MHz, DMSO-d_6) δ 9.44 (t, *J* = 5.3 Hz, 1H), 8.45 (d, *J* = 2.3 Hz, 1H), 8.14 (s, 1H), 7.81 (dd, *J* = 8.3, 2.5 Hz, 1H), 7.55 (d, *J* = 8.2 Hz, 1H), 5.69 (s, 2H), 4.54 (d, *J* = 5.5 Hz, 2H). ^{13}C NMR (100 MHz, DMSO-d_6) δ 156.55, 150.09, 149.47, 144.01, 139.55, 131.34, 124.39, 123.33, 49.38, 34.88. HR-MS (ESI): calcd for $C_{16}H_{10}ClF_5N_5O$, $[M+H]^+$ 418.0494; found: 418.0497. Purity: 98.24%.

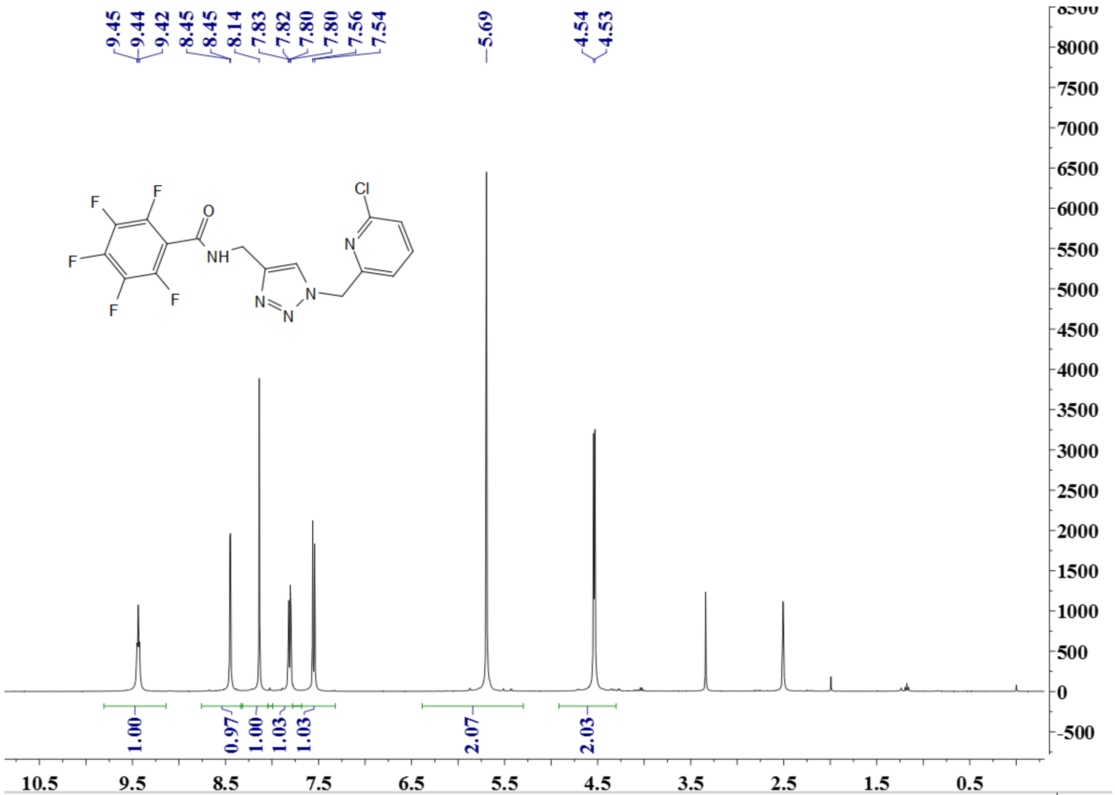

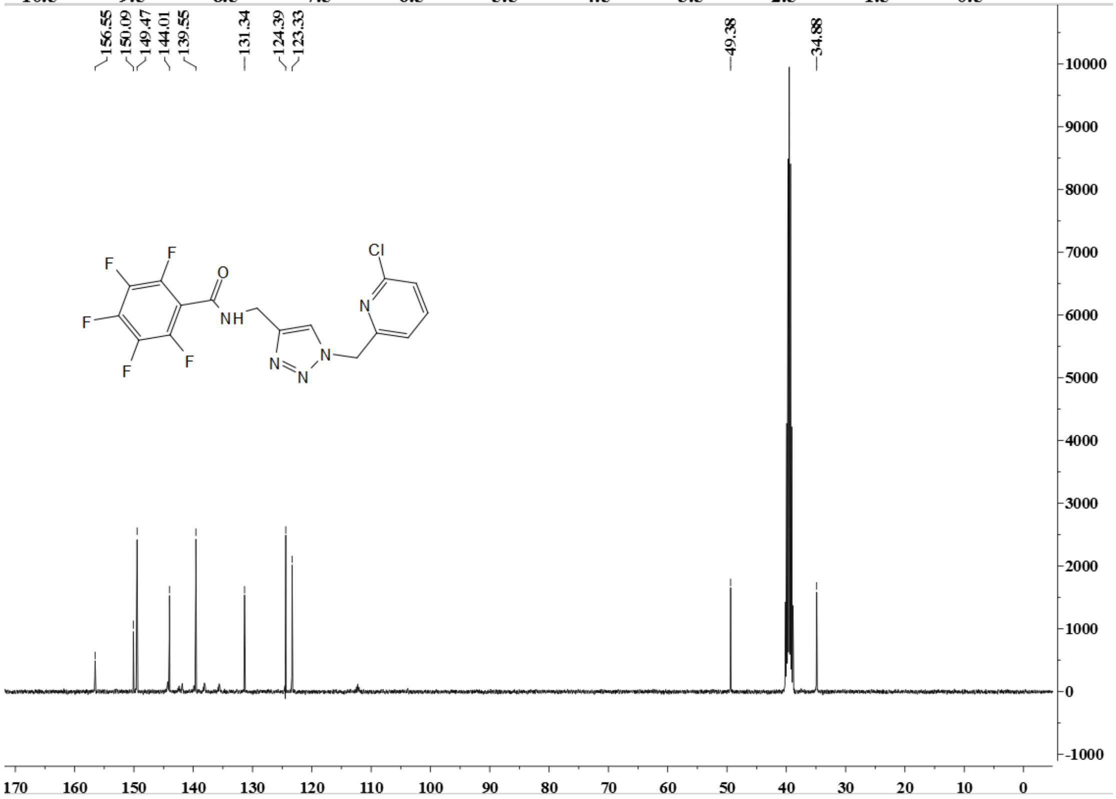

N-((1-((1H-benzo[d]imidazol-2-yl)methyl)-1H-1,2,3-triazol-4-yl)methyl)-2,3,4,5,6-pentafluorobenzamide (C8)

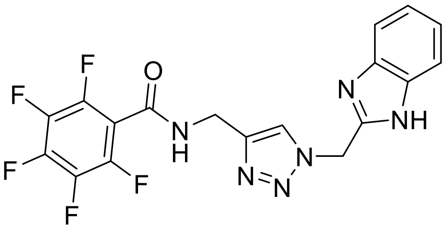

White solid, yield: 73%. Mp: 232-234 °C. ^{1}H NMR (400 MHz, DMSO-d_6) δ 12.68 (s, 1H), 9.46 (t, J = 5.4 Hz, 1H), 8.13 (s, 1H), 7.55 (dd, J = 35.7, 7.6 Hz, 2H), 7.34 – 6.97 (m, 2H), 5.88 (s, 2H), 4.57 (d, J = 5.6 Hz, 2H). ^{13}C NMR (100 MHz, DMSO-d_6) δ 156.59, 148.24, 143.84, 123.66, 47.24, 34.89. HR-MS (ESI): calcd for $C_{18}H_{12}F_5N_6O$, $[M+H]^+$ 423.0993; found: 423.0998. Purity: 98.15%.

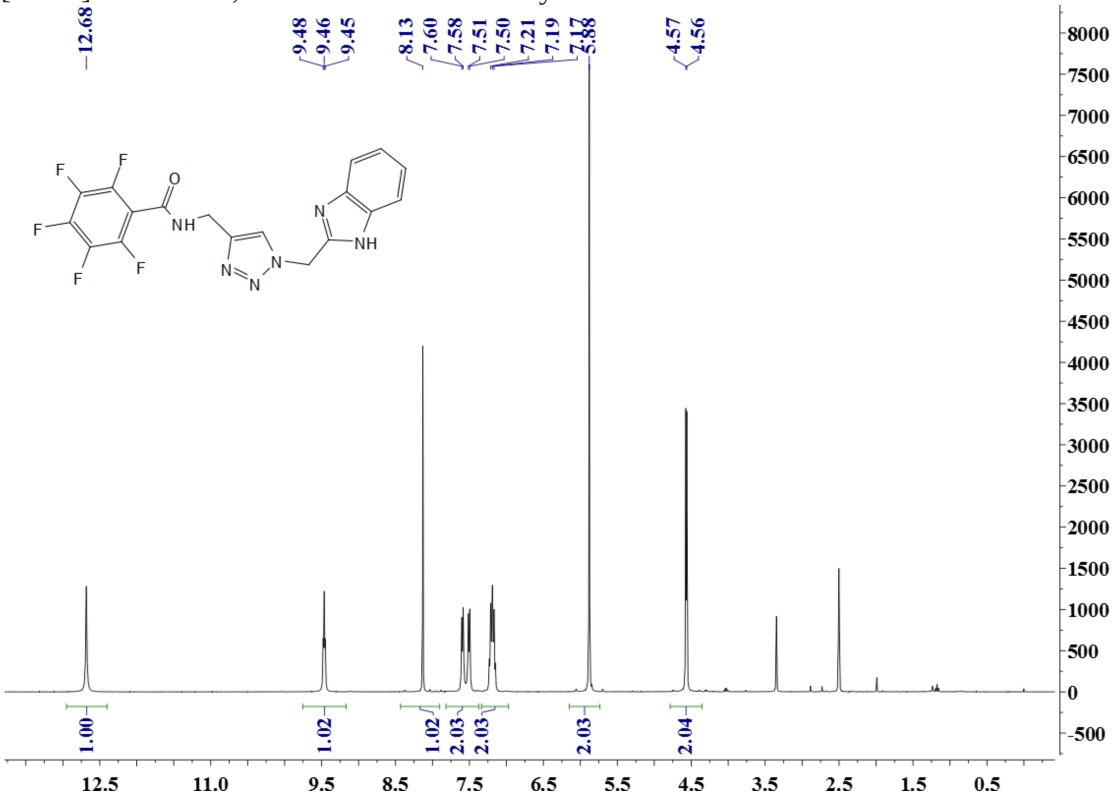

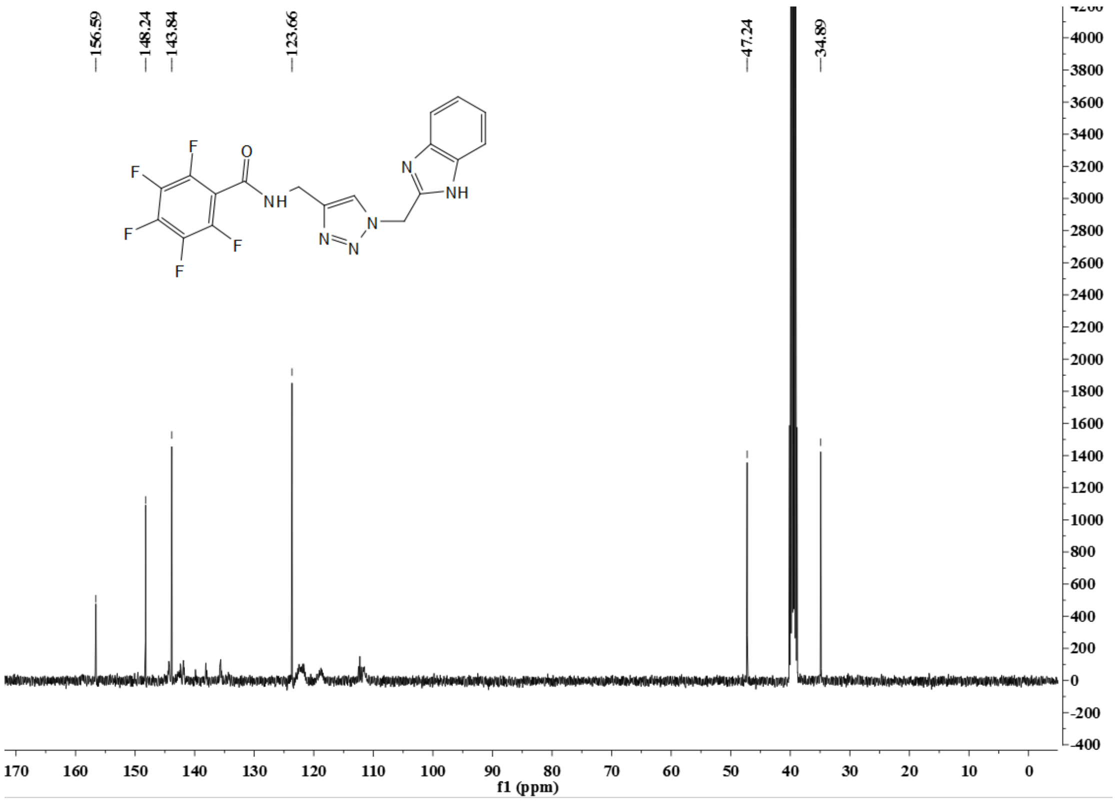

156.59
148.24
143.84
123.66
47.24
34.89
F
F
F
F
F
O
NH
N
N
N
N
NH
f1 (ppm)
